# Gut Microbiota‐Non‐Coding RNA Axis in Immune Modulation and Disease: From Mechanisms to Clinical Translation

**DOI:** 10.1002/advs.202519949

**Published:** 2026-02-06

**Authors:** Bonan Chen, Guoming Chen, Xieyuan Leng, Qianfan Li, Wenhao Wu, Wenqiu Wu, Zixuan Liu, Zilan Zhong, Xiaohong Zheng, Wei Kang, Fazheng Ren, Yigan Zhang, Juan Chen

**Affiliations:** ^1^ Institute of Biomedical Research Department of Infectious Diseases Regulatory Mechanism and Targeted Therapy for Liver Cancer Shiyan Key Laboratory Hubei Provincial Clinical Research Center for Precise Diagnosis and Treatment of Liver Cancer Taihe Hospital Hubei University of Medicine Shiyan Hubei China; ^2^ Hubei Key Laboratory of Embryonic Stem Cell Research Taihe Hospital Hubei University of Medicine Shiyan Hubei China; ^3^ Department of Anatomical and Cellular Pathology State Key Laboratory of Translational Oncology Sir Y.K. Pao Cancer Center The Chinese University of Hong Kong Hong Kong SAR China; ^4^ Institute of Digestive Disease State Key Laboratory of Digestive Disease Li Ka Shing Institute of Health Science The Chinese University of Hong Kong Hong Kong SAR China; ^5^ CUHK‐Shenzhen Research Institute Shenzhen China; ^6^ School of Chinese Medicine Li Ka Shing Faculty of Medicine The University of Hong Kong Hong Kong SAR China; ^7^ The First Clinical College of Guangzhou University of Chinese Medicine Guangzhou China; ^8^ Key Laboratory of Precision Nutrition and Food Quality Department of Nutrition and Health China Agricultural University Beijing China; ^9^ Guangzhou University of Chinese Medicine Guangzhou China; ^10^ The Fourth Clinical Medical College of Guangzhou University of Chinese Medicine Shenzhen China

**Keywords:** clinical applications, gut microbiota, immune regulation, microbial metabolites, non‐coding RNA

## Abstract

Immune homeostasis is indispensable for preserving organismal integrity, orchestrated through complex molecular networks encompassing immune cell dynamics, microbial cues, and epigenetic regulation. Among these, the gut microbiota‐non‐coding RNA (ncRNA) axis has recently garnered substantial attention as a multifaceted modulator of host immunity. Emerging evidence indicates that microbial‐derived metabolites can reprogram ncRNA expression, thereby modulating immune cell differentiation, activation, and effector responses. Notably, dysregulation of this axis has been mechanistically implicated in the etiology of diverse immune‐related pathologies, including colorectal cancer, sepsis, atherosclerosis, and neuroimmune conditions. Particularly intriguing is its translational potential: both microbial signatures and ncRNA profiles are being leveraged as diagnostic biomarkers and actionable targets for immune modulation. In this review, we delineate the molecular frameworks underpinning the gut microbiota‐ncRNA‐immune and explore how its perturbation contributes to pathogenesis. We further highlight emerging therapeutic strategies targeting this axis, underscoring its significance in precision immunology and host‐microbiota co‐regulation.

## 1 Introduction

Immune homeostasis refers to a balanced physiological state that maintains overall health by coordinating protective immune responses with tolerance to self [1, 2]. This balance relies on complex regulatory mechanisms that enable effective defense against external antigens while preventing excessive or misdirected inflammation. Perturbation of immune homeostasis contributes to autoimmune disease, graft rejection, and impaired responses to cancer therapy, reflecting its importance across diverse clinical conditions [3, 4]. These regulatory processes operate within a dynamic cellular environment characterized by continuous renewal throughout multiple tissues [5, 6, 7].

The gut microbiota functions as an integral component of the host environment and exerts major influence on immune development and regulation [8, 9]. The intestinal microbial community supplies metabolic and structural signals that contribute to the maturation of immune cells, the preservation of epithelial integrity, and the calibration of inflammatory responses [10, 11, 12, 13, 14]. Microbial metabolites such as short‐chain fatty acids (SCFAs) and tryptophan derivatives, as well as microbial‐associated molecular patterns, activate specific signaling pathways that shape both innate and adaptive immunity [15, 16, 17, 18]. Disruption of this microbial environment can perturb immune balance and promote inflammatory and metabolic disorders, highlighting the close relationship between microbial states and immune regulation [19, 20, 21].

Among the multiple pathways through which the gut microbiota influences host physiology, including metabolite production, pattern recognition receptor signaling, cytokine networks, and neuroendocrine circuits, non‐coding RNAs (ncRNAs) occupy a distinctive position as integrators of these diverse inputs [22]. Positioned between transcriptional programs and protein effector networks, ncRNAs constitute a regulatory layer that is highly cell‐type specific yet dynamically regulated [23]. Many ncRNA species can also be detected as stable molecules in blood, stool, and extracellular vesicles. These properties make microbiota‐regulated ncRNAs particularly attractive as integrative readouts of microbial and immune states and as sequence‐specific candidates for therapeutic modulation. In parallel, ncRNAs have been recognized as important regulators of immune responses [24, 25, 26, 27]. MicroRNAs (miRNAs), long non‐coding RNAs (lncRNAs), and circular RNAs (circRNAs) participate in transcriptional and post‐transcriptional control of immune cell differentiation, cytokine production, and inflammatory resolution [28, 29, 30, 31]. Aberrant ncRNA expression has been linked to autoimmune diseases, chronic inflammation, malignancy, and metabolic dysfunction [32, 33, 34, 35, 36]. These molecules form a regulatory layer that integrates upstream signals into precise immune outcomes and therefore provides a critical bridge between environmental inputs and cellular responses [37, 38, 39, 40].

**FIGURE 1:**
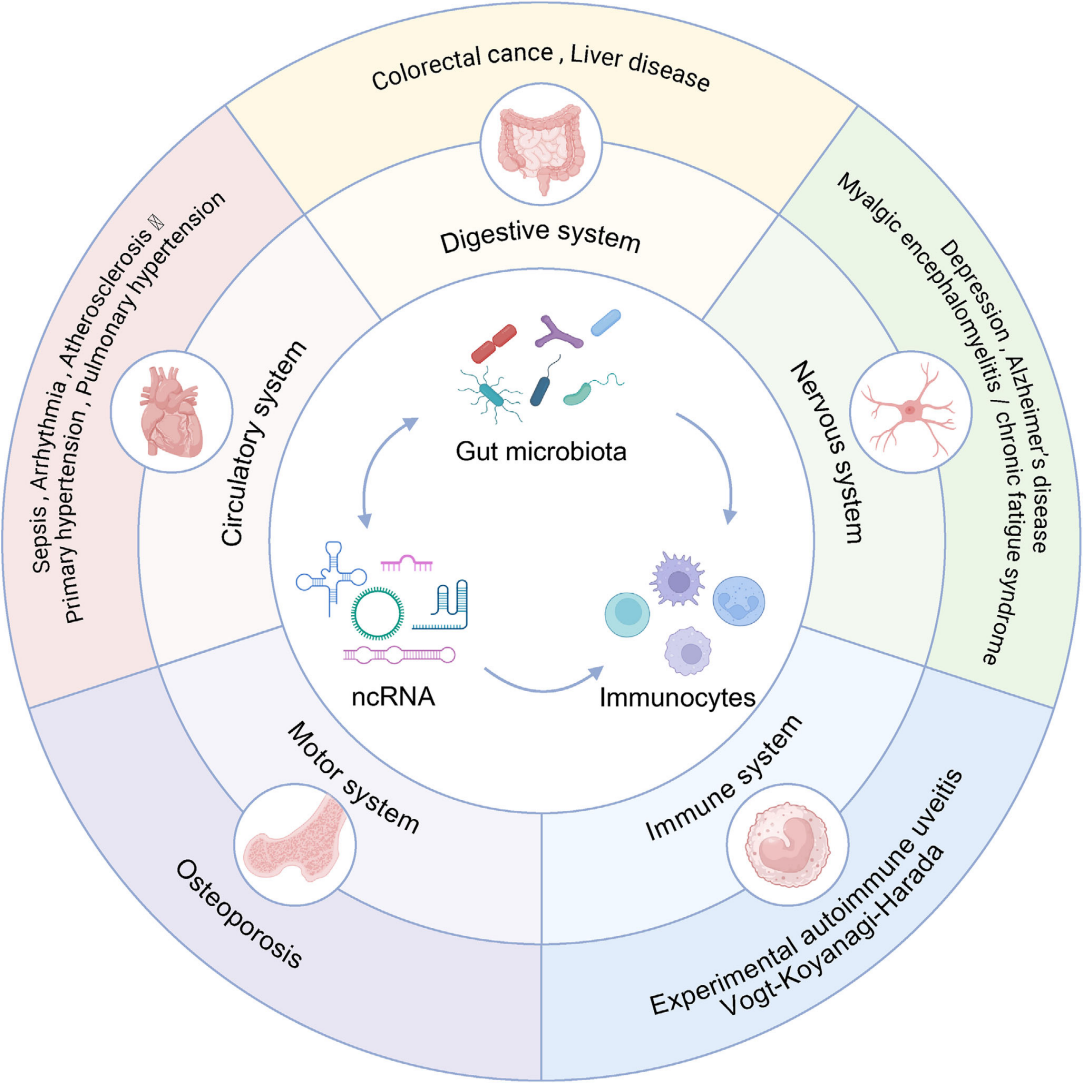
The gut microbiota‐ncRNA axis in disease pathogenesis across systems. The diagram illustrates the role of the gut microbiota and ncRNAs in modulating immune responses and influencing disease progression in various physiological systems. Interactions between the gut microbiota, ncRNAs, and immune cells are implicated in diseases affecting the digestive system (e.g., colorectal cancer, liver disease), circulatory system (e.g., sepsis, primary hypertension), nervous system (e.g., depression, Alzheimer's disease), motor system (e.g., osteoporosis), immune system (e.g., autoimmune diseases), and the nervous system (e.g., myalgic encephalomyelitis/chronic fatigue syndrome).

Recent evidence indicates that the gut microbiota can modulate ncRNA expression, forming a microbiota ncRNA axis that links microbial metabolism to host regulatory programs [41, 42, 43]. Multiple disorders have now been linked to this axis [44, 45, 46]. Through this axis, microbial metabolites and molecular cues modify ncRNA patterns in epithelial, myeloid, and lymphoid cells, leading to changes in gene regulation and immune activity [47, 48, 49]. This concept offers a mechanistic explanation for how variations in microbial composition translate into altered cellular behavior and immune outcomes and provides a framework for exploring the molecular basis of host‐microbe interactions. While previous reviews have summarized the general interactions between gut microbiota and ncRNAs, they primarily focus on descriptive associations and microbiota‐based interventions. In contrast, our review emphasizes the immunological mechanisms driven by the microbiota‐ncRNA axis, particularly in regulating immune cell differentiation, activation, and function via microbial metabolites and signaling pathways. We further highlight its translational relevance in multiple systemic diseases, with special attention to diagnostic and therapeutic potential. This review aims to elucidate the mechanistic basis of gut microbiota‐ncRNA interactions in immune regulation, with a focus on their translational potential in systemic diseases (Figure 1). Furthermore, many ncRNAs have been identified as potential molecular biomarkers or therapeutic targets in colitis‐associated cancers (CACs), offering new avenues for diagnostic and therapeutic innovation.

## 2 Immune‐Regulatory Mechanisms of Gut Microbiota‐ncRNA Interactions

**FIGURE 2:**
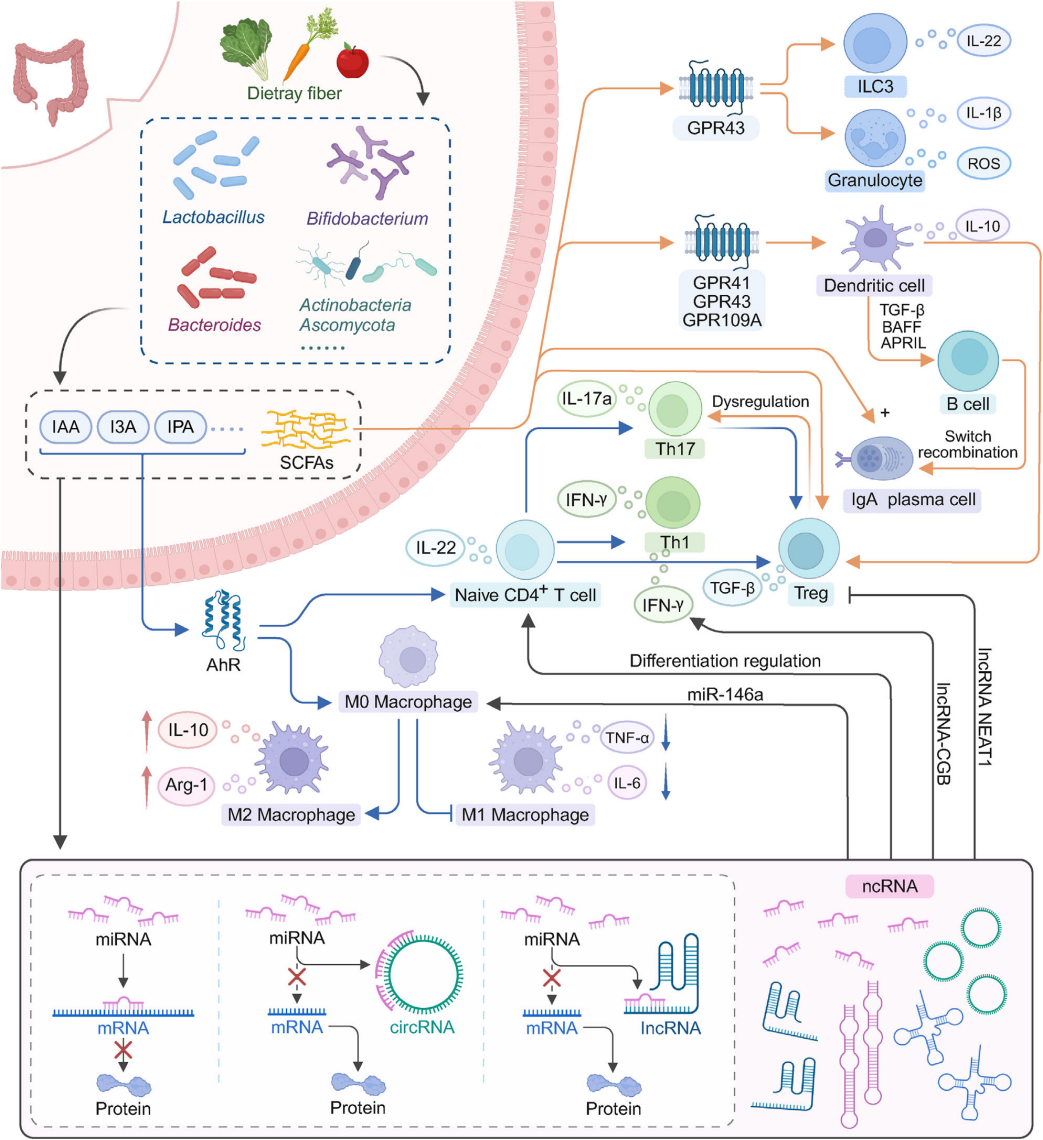
Mechanisms of the gut microbiota‐ncRNA axis in maintaining immune homeostasis. Gut microbiota metabolize dietary fiber into SCFAs (e.g., acetate, propionate, butyrate) and tryptophan derivatives (e.g., I3A, IPA, IAA), which bind to receptors such as GPR41/43 and AhR on immune cells. These signals induce cytokine secretion (e.g., IL‐22, IL‐10) by ILC3s, granulocytes, and DCs, shaping mucosal immunity. Importantly, these microbial metabolites regulate host ncRNA expression—upregulating or downregulating specific miRNAs, lncRNAs, and circRNAs—which in turn modulate immune cell differentiation and cytokine production. Through this multilayered signaling network, the gut microbiota‐ncRNA axis orchestrates Th17/Treg balance and systemic immune homeostasis.

The interactions between gut microbiota, ncRNAs, and the immune system are essential for maintaining immune balance [50]. The gut microbiota communicates with the immune system through microbial metabolites and immune receptor interactions, while ncRNAs regulate immune responses by influencing the differentiation and function of immune cells [51, 52]. Together, this complex network of interactions helps to ensure proper immune function and prevent dysregulation, which is crucial for health (Figure 2). Disruptions in this system can contribute to various inflammatory and autoimmune diseases [53, 54, 55].

### 2.1 Crosstalk Between the Gut Microbiota and the Immune System

The gut microbiota is a complex microbial ecosystem predominantly composed of bacteria, along with smaller populations of archaea, viruses, and fungi, all of which exert profound influences on host physiology, metabolism, and disease susceptibility [56, 57, 58]. In the human gastrointestinal tract, bacteria are the most abundant and diverse, with two dominant phyla: Firmicutes and Bacteroidetes [59, 60]. The phylum Firmicutes includes beneficial genera such as *Lactobacillus* and *Bifidobacterium*, which are mainly involved in carbohydrate fermentation and the production of SCFAs [61]. *Bacteroidetes*, exemplified by the Bacteroides genus, play key roles in degrading dietary polysaccharides and proteins, thereby assisting the host in extracting energy from fiber‐rich diets [62, 63]. *Bacteroides fragilis* upregulates the lncRNA‐CGB in T cells, relieving EZH2‐mediated H3K27me3 repression at the *Ifng* locus and thereby enhancing IFN‐γ‐dependent protection against tuberculosis [42]. Additional members of the gut microbiota include lesser‐abundant phyla, such as Ascomycota and Actinobacteria, as well as species like *Micrococcus luteus* [64, 65, 66]. These microbial communities closely interact with the host immune system throughout life, profoundly shaping its development and functional capacity [67]. Following birth, the gut microbiota rapidly colonizes the host and becomes essential for immune system maturation [68, 69]. Through the release of metabolites such as SCFAs and immune‐modulatory cytokines, gut microbes stimulate the development of the intestinal mucosal immune system and promote immune cell differentiation and maturation [70, 71]. Moreover, the gut microbiota communicates with host immune cells primarily through pattern recognition receptors (PRRs) [72, 73, 74]. For instance, microbial components such as lipopolysaccharide (LPS) and flagellin are recognized by Toll‐like receptors (TLRs), triggering immune activation in cells like macrophages and dendritic cells [75, 76]. In addition, microbial metabolites, including those of tryptophan derivatives, modulate immune activity and help suppress excessive inflammation [77].

These interactions between gut microbiota and the immune system have profound immunomodulatory effects that influence both local and systemic immune responses. The gut microbiota enhances the integrity of the intestinal mucosal barrier by releasing SCFAs and interacting with host epithelial cells. These metabolites stimulate epithelial cell proliferation and repair, thereby reinforcing mucosal barrier function and limiting the entry of pathogens and toxins [78, 79, 80]. Secondly, the gut microbiota regulates immune responses by modulating immune cell activity and function [81, 82]. For example, SCFAs promote the secretion of interleukin‐22 (IL‐22) from CD4^+^ T cells and ILCs [83, 84]. This process involves the GPR41 signaling axis, histone modifications, and regulation via the AhR and hypoxia‐inducible factor‐1α (HIF‐1α) [85]. SCFA supplementation increases IL‐22 production, maintains mucosal integrity, and prevents excessive inflammation, thereby supporting immune homeostasis [83]. Thirdly, microbial influences extend beyond the gut, affecting systemic immunity through blood circulation and neuroendocrine pathways [86, 87]. Gut microbes modulate immune metabolism and memory, impacting pathogen resistance and vaccine responsiveness [88]. Finally, gut microbiota dysregulation—such as reduced diversity or overgrowth of pathogenic taxa—is strongly associated with multiple diseases, including inflammatory bowel disease (IBD), obesity, metabolic syndrome, allergies, and autoimmune disorders [89, 90, 91]. In IBD, for instance, the gut microbiota is characterized by increased levels of potentially harmful bacteria (e.g., certain Ascomycota species) and reduced abundance of beneficial microbes like *Bifidobacterium* [92, 93]. These changes in microbiota composition disrupt immune function, accelerating disease progression and highlighting the critical need for maintaining a balanced gut ecosystem for overall health [94].

### 2.2 Crosstalk Between ncRNAs and the Immune System

ncRNAs are a class of nucleic acids that do not encode proteins but exhibit significant functional and regulatory versatility [95, 96]. They encompass a broad spectrum of RNA molecules with distinct biological functions, including ribosomal RNA (rRNA), transfer RNA (tRNA), small nuclear RNA (snRNA), small nucleolar RNA (snoRNA), and miRNA, as well as several RNAs whose functions remain incompletely defined [97]. In recent years, miRNAs, lncRNAs, and circRNAs have gained increasing attention as newly characterized members of the ncRNA family; however, their precise regulatory roles and mechanisms remain to be fully elucidated [98, 99]. Despite their lack of protein‐coding potential, ncRNAs are essential in regulating gene expression, cellular differentiation, and developmental processes [100]. For example, miRNAs regulate gene expression post‐transcriptionally by binding to complementary sequences within the 3’ untranslated regions (UTRs) of target mRNAs, leading to translational repression or mRNA degradation [101, 102]. LncRNAs, a structurally and functionally heterogeneous group of transcripts, participate in various epigenetic regulatory pathways, including DNA methylation, histone modification, and miRNA interaction [103, 104]. CircRNAs, which are characterized by covalently closed loop structures, modulate gene expression by influencing transcription or splicing and interacting with RNA‐binding proteins to regulate mRNA translation [105, 106].

NcRNAs serve as crucial regulators of cellular functions across various immune cell lineages, from hematopoietic stem cells (HSCs) to innate and adaptive immune cells in humans and mice [107, 108]. Immune‐specific ncRNAs influence hematopoietic differentiation through multiple mechanisms, including functioning as decoys for proteins or other RNAs, or acting within the nucleus and cytoplasm as scaffolds, transporters, and molecular recruiters [109, 110, 111]. As part of innate immunity, macrophages and dendritic cells are particularly sensitive to ncRNA‐mediated regulation, where ncRNAs fine‐tune cytokine release, inflammasome activation, and tissue repair. During immune activation, miRNAs can regulate T cell differentiation and activation by modulating pathways such as RB1/NF‐κB p65, PI3K/AKT, PD‐1/PD‐L1, and JAK2/STAT1 [112]; lncRNAs influence B cell differentiation and activation through transcriptional and post‐transcriptional interactions with DNA, RNA, and proteins [113]; circRNAs regulate T cell development and function by acting as miRNA sponges or interacting with immune‐related proteins [114]. These compartment‐specific regulatory mechanisms extend across both innate and adaptive immunity and underpin ncRNA‐dependent control of regulatory and effector function in diverse immune subsets, including macrophages, dendritic cells, CD4^+^ T cells, CD8^+^ T cells, and B cells.

Substantial progress has been made in understanding the immunoregulatory roles of ncRNAs in inflammatory diseases [115, 116, 117]. In the context of IBD, accumulating evidence demonstrated that miR‐223 modulates inflammation via the TNFα/NFκB/NLRP3 axis, and suppression of NLRP3 expression yields protective effects [118], while additional mechanistic analyses indicated that macrophage‐derived exosomal miR‐223 regulates the intestinal barrier by targeting TMIGD1 [119]. In rheumatoid arthritis (RA), recent findings showed that lncRNA MEG3 upregulates SMAD7 expression by competitively binding to miR‐93‐5p, thereby attenuating proliferation and inflammatory responses in RA synovial fibroblasts [120]. Conversely, several studies also document ncRNAs that promote inflammatory injury. For example, evidence indicates that lncRNA AC006129.1 exacerbates renal ischemic damage by amplifying CXCL2‐mediated inflammation [121], and that lncRNA GUSBP11 intensifies LPS‐induced inflammatory signaling and fibroblast proliferation by downregulating miR‐185‐5p, contributing to chronic periodontitis pathogenesis [122]. Additionally, ncRNAs have garnered attention for their roles in tumor microenvironment (TME) regulation. miRNAs modulate CD8^+^ T cell activation, recruitment, infiltration, effector functions, and TME homeostasis across various cancers [123]. LncRNAs interact with both cellular and non‐cellular components of the TME, facilitating tumor growth, metastasis, drug resistance, and immune evasion [124]. Similarly, circRNAs contribute to TME remodeling by modulating immune‐related gene expression and inflammatory signaling pathways [125]. Collectively, these findings demonstrate that ncRNAs operate across multiple immune compartments and provide a mechanistic foundation for their broad involvement in systemic immune regulation and disease pathogenesis.

### 2.3 Crosstalk Among Gut Microbiota, ncRNAs, and the Immune System

The gut microbiota ncRNA axis plays an essential role in maintaining immune homeostasis by linking microbial metabolites, host RNA regulatory programs, and immune cell function [126, 127]. The gut microbiota communicates with the immune system primarily through structurally conserved ligands and bioactive metabolites such as SCFAs, tryptophan derivatives, and secondary bile acids [128]. These signals not only affect local immune responses in the gut but also influence systemic immunity, helping to maintain a balanced immune state throughout the body [129, 130]. In this context, ncRNAs act as key molecular intermediates that translate microbial cues into cell‐type‐specific transcriptional and post‐transcriptional programs [131, 132, 133]. Their effects are particularly important in regulating the differentiation, activation, and memory of immune cells, which are vital for the maintenance of immune tolerance and the prevention of excessive inflammation [134, 135, 136, 137]. Several mechanistic examples illustrate how microbial metabolites regulate ncRNA expression in defined immune compartments. SCFAs produced by gut microbes have been shown to regulate the expression of miRNAs, such as miR‐223, which plays a critical role in inflammatory responses [138, 139]. In the case of IBD, the downregulation of miR‐223 exacerbates inflammation by promoting the activation of pro‐inflammatory pathways such as TNFα/NFκB and NLRP3 inflammasome signaling [140, 141, 142]. In parallel, microbiota‐derived tryptophan metabolites activate aryl hydrocarbon receptor signaling in innate lymphoid cells and T cells, leading to ncRNA‐dependent regulation of IL‐22 production and modulation of the Th17 Treg balance, which is essential for mucosal defense and tolerance [143]. Microbiota‐regulated lncRNAs and circRNAs also influence immune cell differentiation and function [144]. For example, lncRNAs have been shown to modulate the differentiation of B and T cells by interacting with chromatin or recruiting specific proteins that influence transcriptional activity [145, 146]. Additionally, circRNAs, which act as molecular sponges for miRNAs, can impact immune cell function by regulating immune‐related gene expression, thereby influencing systemic immune responses [40, 147, 148]. Through these pathways, defined microbial metabolites drive ncRNA remodeling in specific immune cell subsets and shape the magnitude and quality of immune responses.

The influence of the gut microbiota on ncRNA expression extends beyond mucosal sites to systemic immunity [149]. SCFAs can reach peripheral tissues and interact with immune cells in secondary lymphoid organs, where they affect T cell activation, antibody production, and memory formation through ncRNA‐mediated mechanisms [129, 150]. These metabolites, along with their effects on ncRNA‐mediated regulation of immune responses, impact pathogen resistance, vaccine responsiveness, and immune memory [151, 152, 153]. For instance, gut microbiota‐derived metabolites have been found to influence T cell activation, thereby enhancing immune responses to infections and vaccinations [154, 155]. In autoimmune conditions, dysregulation of the microbiota‐ncRNA axis can lead to persistent immune activation, as seen in diseases like IBD and RA [156, 157, 158]. In RA, lncRNA MEG3 has been shown to upregulate SMAD7 expression by competing with miR‐93‐5p, which attenuates inflammatory responses in synovial fibroblasts [158]. These findings underscore the significance of the gut microbiota‐ncRNA axis in maintaining immune balance and preventing immune dysregulation.

Importantly, the regulatory relationship between ncRNAs and the gut microbiota is reciprocal. A seminal study demonstrated that host‐derived fecal miRNAs released from intestinal epithelial cells can be taken up by gut bacteria, where they modulate bacterial gene expression, growth, and community structure, providing direct evidence that ncRNAs enable the host to shape microbiota composition [159]. Building on this concept, subsequent work has shown that small RNA fractions isolated from murine feces alter the structure of cultured gut microbiota, and that stool miRNA profiles in humans are associated with distinct microbial configurations and may directly influence bacterial gene expression and proliferation [160, 161]. Furthermore, extracellular vesicle‐associated miRNAs have been proposed as a major route for this inter‐kingdom RNA transfer, further supporting a bidirectional ncRNA‐mediated communication axis between host and microbiota [162, 163]. In addition to such direct RNA exchange, host ncRNAs can influence microbial ecology indirectly by controlling mucosal barrier integrity and the chemical environment of the intestinal lumen [164, 165]. Multiple miRNAs and lncRNAs regulate tight junction proteins, mucus layer components, and antimicrobial peptides in intestinal epithelial cells, thereby affecting epithelial permeability and the ecological niches available for commensal and pathogenic bacteria [166, 167]. By shaping secretory IgA responses and cytokine and chemokine profiles in mucosal B and T cells, ncRNAs further modulate the selective pressure acting on different microbial taxa, providing an additional route through which host RNA networks can alter microbiota composition and function [168, 169].

In conclusion, the gut microbiota‐ncRNA axis plays a critical role in immune homeostasis by modulating immune cell differentiation, activation, and systemic immune responses. Through microbial metabolites such as SCFAs and direct interaction with ncRNAs, gut microbes influence both local and systemic immunity, contributing to the regulation of inflammatory responses and immune memory [170, 171, 172]. Disruptions in this delicate network, such as changes in microbiota composition or dysregulated ncRNA expression, can lead to the development of inflammatory and autoimmune diseases, highlighting the importance of maintaining a balanced gut microbiota for immune health [173, 174, 175].

## 3 Disease Contexts of the Gut Microbiota‐ncRNA Axis

The gut microbiota‐ncRNA axis is increasingly recognized as a crucial player in the regulation of immune homeostasis, with profound implications for disease pathogenesis. Dysregulation of this axis has been implicated in a wide range of diseases, where microbial‐induced changes in ncRNA expression contribute to alterations in immune cell function, differentiation, and activation. Understanding the mechanisms through which the gut microbiota influences ncRNA expression provides insights into the multifaceted roles these interactions play in diseases affecting various organ systems. From the digestive system to the nervous system, the gut microbiota‐ncRNA axis impacts disease development, progression, and therapeutic outcomes. In this section, we explore the distinct mechanisms of the microbiota‐ncRNA axis in different disease contexts, focusing on its immunological roles and the implications for potential therapeutic interventions.

### 3.1 Digestive Diseases

#### 3.1.1 Colorectal Cancer

**FIGURE 3:**
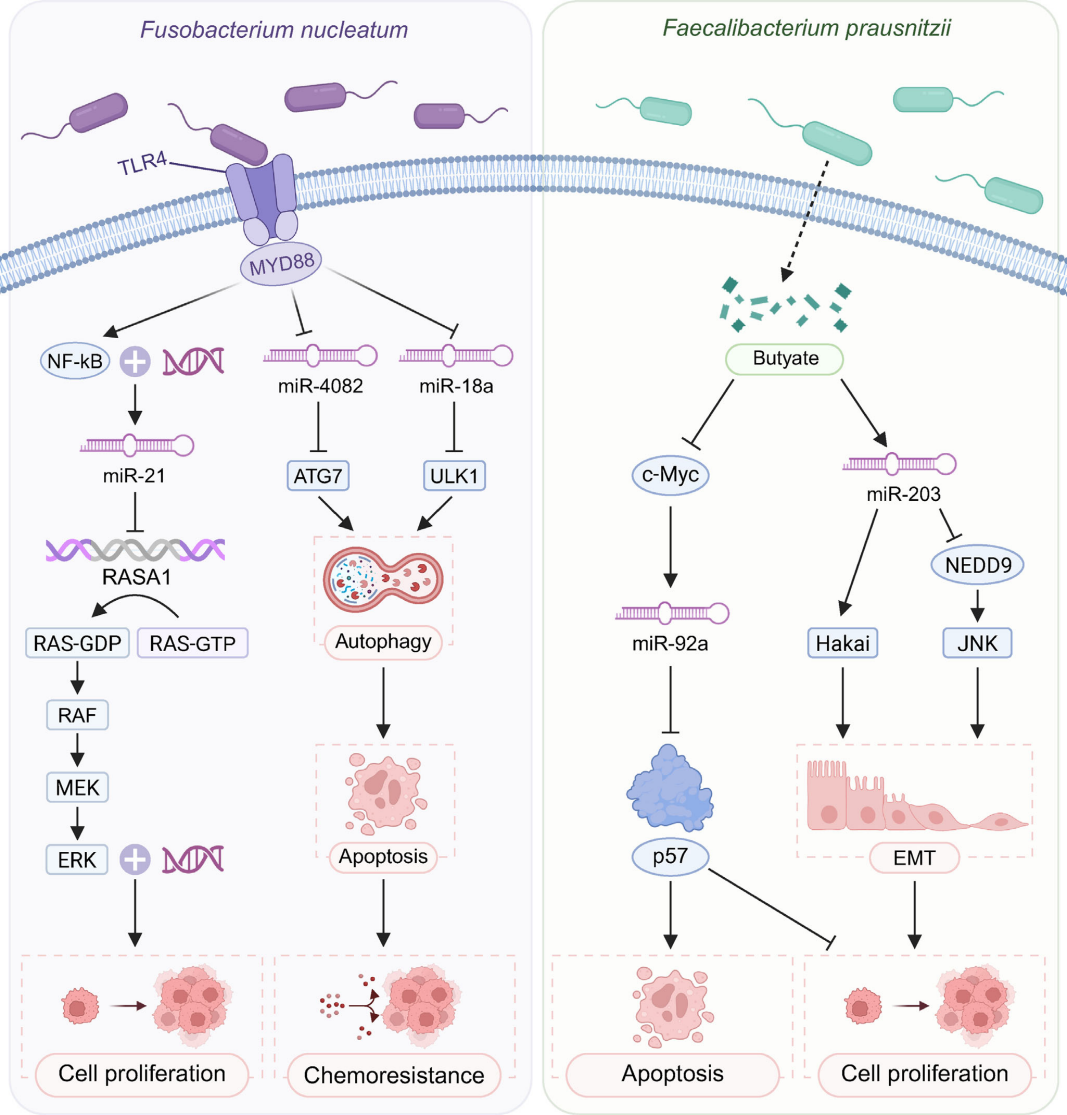
Gut microbiota‐derived modulation of ncRNAs in colorectal cancer pathogenesis. *F. nucleatum* activates TLR4‐MYD88 signaling in host cells, promoting NF‐κB‐mediated transcription of oncogenic miR‐21, which suppresses RASA1 and activates the RAS‐RAF‐MEK‐ERK cascade, thereby enhancing cell proliferation. Concurrently, *F. nucleatum* downregulates miR‐4802 and miR‐18a, relieving inhibition of autophagy‐related genes ATG7 and ULK1, leading to increased autophagy and chemoresistance. In contrast, *F. prausnitzii* produces butyrate, which downregulates c‐Myc and subsequently miR‐92a, resulting in p57 upregulation and enhanced apoptosis. Butyrate also induces miR‐203, which suppresses epithelial‐EMT and cell proliferation by targeting Hakai and JNK signaling via NEDD9.

Colorectal cancer (CRC) is closely associated with the gut microbiota, wherein ncRNAs play a pivotal regulatory role [149, 176]. A growing body of evidence suggests that the gut microbiota can modulate the expression of host ncRNAs, thereby influencing tumor initiation, progression, and immune surveillance [177, 178, 179]. Specifically, certain microbial taxa are capable of activating or repressing distinct ncRNAs, orchestrating critical cellular processes such as proliferation, apoptosis, and inflammatory responses. This intricate interplay between the microbiota and host ncRNAs offers novel insights and potential therapeutic targets for CRC management. Numerous studies support the close association between CRC progression and the intestinal microbiota, in which miRNAs act as critical mediators [180]. Certain tumor‐related miRNAs are consistently upregulated in the circulation of cancer patients [181, 182, 183]. Among these, miR‐21 stands out as one of the most widely studied oncogenic factors. It promotes colorectal carcinogenesis by targeting PTEN, PDCD4, and DKK2, and by modulating the mitogen‐activated protein kinase (MAPK) and WNT/β‐catenin signaling pathways [184, 185, 186]. Conversely, some miRNAs exhibit tumor‐suppressive functions by downregulating oncogenes involved in proliferation, apoptosis, invasion, and migration [187, 188]. For instance, miR‐34a inhibits CRC metastasis by targeting Notch1/Jagged1, thereby suppressing epithelial‐mesenchymal transition (EMT) markers such as vimentin and fibronectin [189, 190, 191]. Several CRC‐associated bacterial species, such as *Fusobacterium nucleatum* (*F. nucleatum*) and *Faecalibacterium prausnitzii* (*F. prausnitzii*), have been shown to influence miRNA expression in CRC cells, providing a representative model of microbiota‐driven ncRNA regulation with clear links to immune‐related tumor progression that is summarized schematically in Figure 3 [192, 193]. In vitro and mouse studies indicate that *F. nucleatum* can activate the TLR4‐MyD88‐NF‐κB axis, leading to upregulation of miR‐21 and downregulation of miR‐4802 and miR‐18a, thereby suppressing RASA1 and PDCD4 and enhancing ULK1‐ATG7‐dependent autophagy and chemoresistance. Multiple patient cohorts show enrichment of *F. nucleatum* in a subset of CRCs, although the magnitude of association differs across populations and tumor locations, suggesting that this organism contributes within specific dysbiotic communities rather than acting as a solitary driver [194, 195]. Moreover, certain strains of *E. coli* produce colibactin, which causes DNA damage and enhances c‐Myc expression. Experimental studies indicated that colibactin‐producing *E. coli* can induce DNA damage and activate a c‐Myc‐miR‐20a‐5p‐SENP1 axis, promoting senescence‐associated secretory phenotypes and tumor‐like changes in colonic epithelium. In human cohorts, pks^+^ *E. coli* strains are enriched in a subset of colitis‐associated cancers, but their prevalence is heterogeneous across populations, suggesting a context‐dependent contributory role rather than a universal driver [196]. For *P. gingivalis*, current evidence linking oral or intestinal colonisation to CRC relies on limited cohorts and preclinical models in which inflammasome activation and miRNA remodeling promote pro‐tumoral inflammation. These findings suggest a potential contributory role but fall short of establishing *P. gingivalis* as a consistent causal factor in human CRC [197]. Reduced abundance of the butyrate‐producing commensal *F. prausnitzii* has been repeatedly observed in CRC‐associated dysbiosis, and experimental supplementation suppresses c‐Myc‐miR‐92a signaling and tumor growth in mouse models. However, whether *F. prausnitzii* exerts a direct tumor‐suppressive effect in humans or mainly marks a broader loss of anti‐inflammatory consortia remains unresolved [198, 199]. Butyrate also induces miR‐203 expression, which inhibits EMT by downregulating NEDD9 and Hakai via the JNK signaling axis [200]. To provide a comprehensive overview of the immunoregulatory mechanisms linking gut microbiota and host miRNAs, the key interactions are summarized in Table 1 [201, 202, 203, 204, 205, 206, 207, 208, 209, 210].

**TABLE 1:** Immunoregulatory mechanisms of the gut microbiota‐ncRNA axis.

Microorganism	Related miRNA	Target/Mechanism	Effect on immunity	Refs.
*P. aeruginosa*	miR‐302b	Targets IRAK4, inhibits TLR‐induced NF‐κB activation	Attenuates inflammatory exacerbation	[201]
*P. aeruginosa*	miR‐301b	Targets c‐Myb, inhibits expression of anti‐inflammatory cytokines IL‐4 and TGF‐β1	Exacerbates inflammatory responses	[202]
*N. gonorrhoeae*	miR‐718	Targets PTEN, modulates PI3K/AKT signaling	Suppresses pro‐inflammatory cytokine production	[203]
*B. abortus*	miR‐125b‐5p	Downregulates NF‐κB pathway	Suppresses inflammation, enhances bacterial survival	[204]
*M. tuberculosis*	let‐7f	Downregulates NF‐κB pathway	Suppresses inflammation, enhances bacterial survival	[205]
*M. tuberculosis*	miR‐26a‐5p	Reduces KLF4 expression	Promotes M2 macrophage polarization	[206]
*M. tuberculosis*	miR‐223	Reduces IL‐6, CXCL2 and CCL3 expression	Inhibits neutrophil recruitment to infected tissues	[207]
*S. aureus*	miR‐24	Activates CHI3L1‐mediated MAPK pathway	Enhances M1 macrophage polarization	[208]
*S. enterica*	miR‐128	Targets M‐CSF	Inhibits macrophage accumulation at infection sites	[209]
*H. pylori*	miR‐21, miR‐222	Targets tumor suppressors RECK and HIPK2 to inhibit their function	Promotes tumor cell proliferation and invasion	[210]

Beyond miRNAs, emerging studies highlight the role of lncRNAs and circRNAs in microbiota‐host crosstalk [211, 212]. Microbial species, including those associated with CRC, can modulate the expression of lncRNAs and circRNAs, which in turn influence tumorigenic pathways [213, 214]. For example, *F. nucleatum* infection can alter the expression of lncRNAs such as LINC00460, which functions as a competing endogenous RNA (ceRNA) that sponges miR‐186‐3p to upregulate MYC, CD47, and PD‐L1, facilitating immune evasion in CRC. MYC, in turn, transcriptionally activates LINC00460, forming a self‐reinforcing loop that amplifies CRC progression under microbial influence [211]. Similarly, microbial dysbiosis has been shown to regulate the expression of circRNAs, such as circ‐0084615 and circSPARC [215, 216]. For example, dysbiotic consortia enriched in *Bacteroides fragilis* (*B. fragilis*) and *E. coli* have been reported to alter the levels of circ‐0084615 in experimental models; this circRNA acts as a sponge for miR‐599 to regulate DNMT3A expression, thereby promoting CRC cell proliferation and migration [215, 217]. Likewise, circSPARC, influenced by microbial metabolites, sequesters miR‐485‐3p, leading to the activation of the JAK/STAT pathway and increased CRC cell migration [216]. In addition, YY1—a transcription factor regulated by both microbial‐derived signals and host factors—plays a critical role in CRC progression. YY1 suppresses the expression of miR‐500a‐5p, activates Wnt/β‐catenin signaling, and represses p53 expression, collectively promoting tumor growth and metastasis [218, 219]. Microbial regulation of YY1 has been partially elucidated: A recent study demonstrated that *Salmonella enterica* (*S. enterica*) infection induces histone lactylation at the promoter region of the lncRNA LINC00152 in intestinal epithelial cells, thereby reducing YY1 binding efficiency. This epigenetic modification leads to the upregulation of LINC00152, which in turn downregulates IL‐8 and TNF‐α—key targets of the NF‐κB pathway—thereby potentially modulating host immune responses and bacterial internalization [220]. These findings highlight a mechanism by which microbial factors indirectly modulate YY1 activity, and more broadly, how the gut microbiota regulates host lncRNA and circRNA expression. Furthermore, plant‐derived exosome‐like nanoparticles (ELNs) have recently been shown to participate in microbiota‐host communication [221, 222]. These ELNs can be internalized by gut bacteria and carry regulatory RNAs that influence both microbial composition and host physiology [222, 223, 224]. Ginger‐derived ELNs (GELNs), for example, are preferentially taken up by *Lactobacillaceae* via a lipid‐dependent mechanism and deliver miRNAs targeting various genes in *Lactobacillus rhamnosus (LGG)*. One such miRNA, GELN mda‐miR7267‐3p, promotes *LGG* production of monooxygenase YcnE via the microbial metabolite indole‐3‐carboxaldehyde (I3A). Both GELN‐RNAs and I3A stimulate IL‐22 production, enhancing gut barrier function and ameliorating colitis in mice through an IL‐22‐dependent mechanism [222, 225].

Beyond influencing tumor‐intrinsic signaling pathways, microbiota‐regulated ncRNAs also contribute to remodeling the colorectal tumor microenvironment [226, 227]. Several microbial species associated with CRC have been shown to alter ncRNA expression patterns that modulate immune infiltration and local immune suppression [228]. For example, microbiota‐induced ncRNAs that enhance PD‐L1 or CD47 expression can weaken antitumor immunity by limiting cytotoxic T cell activity and promoting macrophage‐mediated immune evasion [211, 229]. In contrast, ncRNAs downregulated under dysbiosis‐associated conditions may impair antigen presentation or T cell recruitment, thereby reshaping the composition and function of immune cells within the colorectal TME [230]. These microbiota‐ncRNA interactions have direct implications for immune checkpoint blockade in CRC. NcRNAs induced by microbial signals can influence pathways linked to T cell exhaustion, PD‐1/PD‐L1 signaling, and interferon responsiveness, potentially affecting sensitivity or resistance to checkpoint inhibitors [231]. Conversely, ncRNAs that promote antigen presentation, enhance CD8^+^ T cell effector programs, or limit immunosuppressive signaling may support more favorable responses to immunotherapy, suggesting that modulation of microbiota‐dependent ncRNA networks could optimize therapeutic outcomes [232, 233].

Emerging evidence further indicates that microbiota‐shaped ncRNAs may serve as clinically informative biomarkers in CRC [234, 235]. Circulating or tumor‐enriched ncRNAs influenced by microbial dysbiosis reflect both tumor biology and local immune states, offering potential utility in diagnosing CRC, predicting disease progression, or assessing responsiveness to immune‐based therapies [236, 237]. Similar patterns of microbiota‐ncRNA regulation have also been observed in certain extraintestinal malignancies, implying that these RNA signatures may capture broader microbiota‐driven immune alterations relevant to cancer development and treatment [214].

#### 3.1.2 Liver Disease

Accumulating evidence suggests that alterations in the gut microbiota can significantly influence hepatic pathophysiology through modulation of ncRNAs, particularly miR‐30a‐5p, thereby establishing a functional “gut‐liver axis” [238, 239, 240, 241] For example, Subsequent research confirmed that gut microbiota derived from miR‐30a‐5p knockout mice could exacerbate hepatic steatosis and arachidonic acid (AA) metabolic disturbances in high‐fat diet (HFD)‐fed wild‐type mice following fecal microbiota transplantation (FMT), confirming a causal role for microbiota in miR‐30a‐5p–mediated liver injury [240, 242, 243]. Shotgun metagenomics and hepatic metabolomic analyses further revealed that microbial dysbiosis influenced the expression of genes involved in AA metabolism, particularly within the cyclooxygenase (COX) and lipoxygenase (LOX) pathways [244, 245]. Notably, increased expression of ALOX5 and ALOX12 enzymes results in enhanced production of pro‐inflammatory and pro‐oxidative lipid mediators, such as 5‐HETE, LTA4, LTB4, LTC4, and 12‐HETE [246, 247]. These metabolites exacerbate hepatic injury by promoting lipid peroxidation, neutrophil recruitment, and cytokine release [248]. Importantly, ALOX12 has been implicated in the initiation of ferroptosis—a form of regulated cell death driven by iron‐dependent lipid peroxidation [249, 250, 251]. In murine models of acute liver injury and ischemia/reperfusion, upregulation of ALOX12 correlates with increased mitochondrial oxidative damage and hepatocyte death [252]. Restoration of miR‐30a‐5p expression attenuates these deleterious effects by suppressing LOX gene expression and reducing lipid peroxidation, thereby preserving mitochondrial integrity and hepatic function. Collectively, these findings highlight miR‐30a‐5p as a central mediator in the gut microbiota‐liver axis. By regulating the AA metabolic pathway and maintaining redox homeostasis, miR‐30a‐5p offers a promising therapeutic target in NAFLD and other liver diseases associated with microbiota dysbiosis.

### 3.2 Cardiovascular and Metabolic Diseases

#### 3.2.1 Sepsis

**FIGURE 4:**
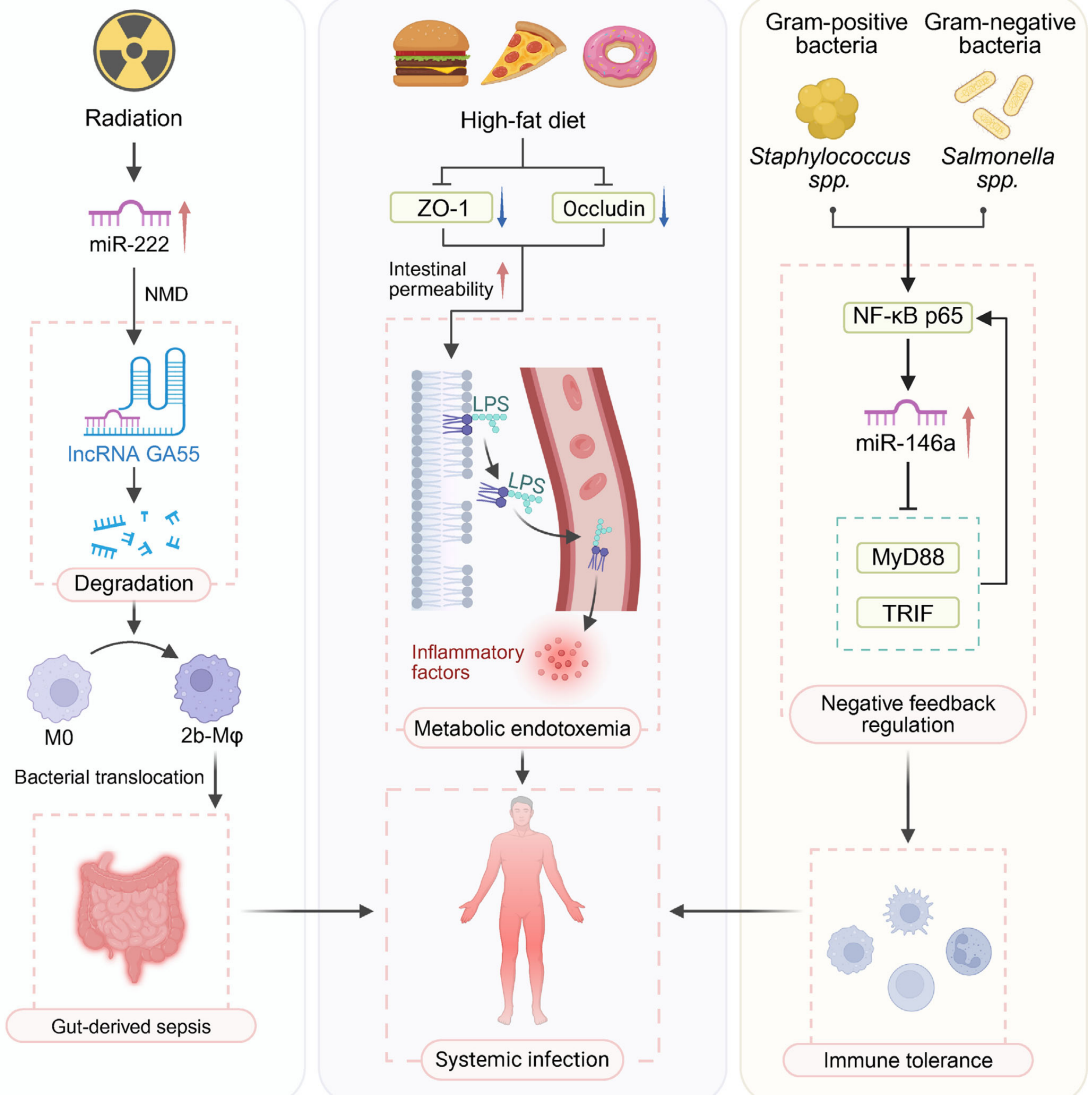
Microbiota‐ncRNA mediated pathways linking gut barrier disruption, systemic infection, and immune tolerance in sepsis. This diagram illustrates how different environmental factors influence the gut microbiota‐ncRNA axis to regulate immune responses. Radiation induces miR‐222 expression, leading to the degradation of lncRNA GA55 and the reprogramming of macrophages (M0 to 2b‐Mφ), resulting in gut‐derived sepsis through bacterial translocation. A high‐fat diet increases intestinal permeability via ZO‐1 and occludin regulation, facilitating the translocation of LPS and triggering inflammatory responses, which contribute to metabolic endotoxemia and systemic infection. Exposure to Gram‐positive bacteria, such as *Staphylococcus spp*., and Gram‐negative bacteria, such as *Salmonella spp*. activates NF‐κB p65 signaling, upregulating miR‐146a and modulating immune tolerance through a negative feedback loop involving MyD88 and TRIF.

Emerging evidence has highlighted the critical role of gut microbiota‐ncRNA interactions in the pathogenesis of sepsis, particularly through modulation of immune responses and epithelial barrier function (Figure 4) [253, 254]. Ionizing radiation has been shown to reprogram macrophage phenotypes by upregulating miR‐222, which in turn promotes the degradation of the lncRNA GAS5 via activation of the nonsense‐mediated mRNA decay (NMD) pathway [255, 256]. This leads to a persistent reduction in GAS5 levels throughout the murine lifespan, thereby rendering mice with the resulting 2b‐Mφ phenotype more susceptible to gut‐derived sepsis triggered by bacterial translocation [257]. Dietary factors such as an HFD can further exacerbate susceptibility to sepsis by altering the gut microbiota composition and impairing intestinal barrier integrity [258, 259, 260, 261]. Specifically, HFD downregulates the expression of tight junction proteins, including zonula occludens‐1 (ZO‐1) and occludin, resulting in increased intestinal permeability and facilitating translocation of LPS into systemic circulation—a process termed metabolic endotoxemia [262, 263, 264]. This translocation initiates robust inflammatory responses and primes the host for systemic infection [265, 266]. Several bacterial species commonly implicated in sepsis, such as *Staphylococcus spp*. and *Salmonella*, have been shown to modulate the inflammatory response by inducing epigenetic reprogramming of innate immune cells [267, 268, 269]. Among the key regulatory ncRNAs, miR‐146a plays a central role in fine‐tuning the TLR signaling cascade [270, 271]. Its transcription is directly upregulated by NF‐κB p65, and miR‐146a subsequently suppresses NF‐κB signaling via targeted degradation of MyD88 and TRIF, forming a negative feedback loop that promotes immune tolerance [256]. While this loop mitigates hyperinflammation, excessive tolerance can impair pathogen clearance and contribute to increased sepsis‐associated morbidity and mortality [272, 273]. Furthermore, the histone demethylase JMJD3 (KDM6B) has been identified as a negative regulator of miR‐146a expression. JMJD3 promotes demethylation of the repressive histone mark H3K27me3 at the miR‐146a promoter, thereby reducing its transcription [274]. Pharmacological inhibition of JMJD3 using the small‐molecule inhibitor GSKJ4 enhances miR‐146a levels, leading to suppressed pro‐inflammatory cytokine expression and improved survival outcomes in murine models of sepsis [256]. These findings underscore the therapeutic potential of targeting ncRNA epigenetic regulators in sepsis and highlight the intricate interplay between the gut microbiota, immune system, and ncRNA signaling.

#### 3.2.2 Arrhythmia

Emerging evidence suggests that the gut microbiota may influence cardiac electrophysiology and arrhythmogenesis through modulation of ncRNAs [275, 276, 277, 278]. These ncRNAs not only mediate host‐microbe communication but also serve as potential biomarkers for microbiota‐associated cardiovascular disorders, including arrhythmias [219]. Dysbiosis of the gut microbiota has been shown to alter host epigenetic landscapes and transcriptional responses, thereby impacting cardiac function [279, 280]. Recent transcriptomic analyses have revealed that several lncRNAs—such as LINC00844, RP11‐532N4.2, and UNC5B‐AS1—are differentially expressed in patients with atrial fibrillation (AF) compared to healthy individuals [281, 282]. These lncRNAs are thought to contribute to arrhythmogenic processes by modulating calcium signaling and TLR pathways [283, 284]. Among miRNAs, miR‐146b‐5p has been identified as a key regulator of atrial fibrosis by influencing the TGF‐β1/Smad3 signaling cascade [285, 286, 287, 288]. This pathway plays a pivotal role in fibrotic remodeling through its impact on matrix metalloproteinases (MMPs) and tissue inhibitors of metalloproteinases (TIMPs), ultimately promoting collagen deposition and structural changes in the atrial myocardium [289, 290, 291]. Additionally, miR‐23a‐3p has been shown to promote ferroptosis by targeting SLC7A11, implicating iron‐dependent cell death as a novel mechanism contributing to AF pathogenesis [292, 293, 294, 295]. Notably, alterations in gut microbiota composition can influence the hepatic expression of miR‐34a [197, 275], which subsequently upregulates the expression of tandem pore domain acid‐sensitive potassium channel 1 (TASK‐1). TASK‐1 activation reduces the atrial resting membrane potential, thereby altering potassium channel conductance and increasing the susceptibility to arrhythmic events [296]. Collectively, these findings underscore the complex regulatory network involving gut microbiota‐derived signals and ncRNA expression in the heart. Targeting this gut‐ncRNA‐heart axis may offer new avenues for the diagnosis and treatment of arrhythmias.

#### 3.2.3 Atherosclerosis

Growing evidence supports the crucial role of gut microbiota‐derived signals in the pathogenesis of atherosclerosis, largely through modulation of ncRNAs that orchestrate lipid metabolism, immune responses, and endothelial function [297, 298]. Several microbiota‐derived metabolites, including SCFAs and indole derivatives, exert regulatory control over ncRNA expression, thereby influencing atherogenic processes [44, 299]. For instance, propionic acid (PA), a SCFA produced by microbial fermentation, suppresses the expression of Niemann‐Pick C1‐like 1 (NPC1L1), a key intestinal cholesterol transporter, through expansion of Tregs and increased interleukin‐10 (IL‐10) production within the intestinal microenvironment [300, 301]. This mechanism alters intestinal cholesterol absorption and contributes to systemic lipid imbalance and atherosclerosis development [302]. In patients with coronary artery disease (CAD), reduced levels of the gut microbiota‐derived metabolite indolepropionic acid (IPA) are associated with dysregulated macrophage responses [303, 304]. Specifically, overexpression of miR‐142‐5p suppresses ATP‐binding cassette transporter A1 (ABCA1), impairing cholesterol efflux to apolipoprotein A‐I (ApoA‐I), and promoting foam cell formation and plaque progression [305, 306]. Similarly, miR‐10b has been identified as a negative regulator of cholesterol efflux via direct targeting of ABCA1 and ABCG1, facilitating lipid accumulation in vascular macrophages [220, 307]. In human cohorts, higher circulating IPA or SCFA levels are associated with lower cardiovascular risk, but the responsible microbial taxa differ between studies, and direct causal links between specific species, ncRNA changes, and clinical atherosclerosis remain to be firmly established. Notably, dietary cyanidin‐3‐glucoside (Cy‐3‐G) is metabolized by gut bacteria into protocatechuic acid (PCA), which suppresses miR‐10b expression and restores ABCA1/ABCG1 levels, thereby enhancing cholesterol efflux and exerting anti‐atherosclerotic effects [308].

In the context of immune regulation, miR‐155 was one of the first miRNAs shown to mediate TLR activation upon LPS stimulation [309, 310, 311]. It promotes pro‐inflammatory cytokine production (e.g., TNF‐α and IL‐6) by repressing SOCS1 and SHIP1, key negative regulators of the JAK/STAT and PI3K/AKT pathways, respectively [312, 313, 314]. Additionally, miR‐181a, which plays a critical role in thymic selection and T‐cell receptor (TCR) sensitivity, intensifies TCR signaling by inhibiting phosphatases during early T‐cell development [315]. Moreover, vascular expression of miR‐204 is under microbiota control and has been shown to impair endothelial function by targeting Sirtuin 1 (Sirt1), a key regulator of oxidative stress and vascular integrity [316]. Butyrate, another major SCFA produced by commensal bacteria, exerts anti‐inflammatory and anti‐atherogenic effects partly via modulation of ncRNAs [317, 318, 319, 320]. Butyrate treatment has been shown to regulate the expression of miRNAs such as miR‐7a‐5p, while also inhibiting histone deacetylase (HDAC) activity, thus suppressing TNF‐α secretion and NF‐κB signaling in monocytes and neutrophils [317, 321]. Additionally, butyrate upregulates IL‐10 expression and enhances peroxisome proliferator‐activated receptor gamma (PPAR‐γ), which acts as an E3 ubiquitin ligase promoting NF‐κB/p65 degradation. These anti‐inflammatory actions are further reinforced by inhibition of miR‐130a, which alleviates vascular inflammation through activation of the PPARγ/NF‐κB signaling axis [317, 322]. Collectively, these findings underscore the multifaceted role of gut microbiota‐ncRNA interactions in the development and progression of atherosclerosis. By shaping lipid handling, immune cell activation, and endothelial function, microbiota‐derived signals and their regulation of ncRNAs offer novel therapeutic opportunities for the management of cardiovascular disease.

#### 3.2.4 Primary Hypertension

Emerging evidence has demonstrated that gut microbiota‐derived signals can modulate host blood pressure homeostasis via epigenetic and post‐transcriptional mechanisms involving ncRNAs, particularly miRNAs [323, 324]. These miRNAs orchestrate critical processes such as vascular tone regulation, renal sodium handling, and systemic inflammation, thereby contributing to the onset and progression of primary hypertension [325, 326]. In hypertensive patients, aberrant expression of specific miRNAs, such as miR‐27a and miR‐27b, has been associated with endothelial dysfunction [327, 328]. These miRNAs are enriched in circulating extracellular vesicles and impair vasodilatory signaling by reducing phosphorylation of endothelial nitric oxide synthase (eNOS) and blunting angiotensin‐(1‐7)‐mediated vascular relaxation [329, 330]. Additionally, they promote abnormal angiogenesis and vascular remodeling, hallmarks of hypertension pathogenesis [323]. Gut microbiota‐derived SCFAs, including acetate, propionate, and butyrate, have been implicated in regulating blood pressure through interactions with SCFA receptors such as GPR41, GPR43, and Olfr78, particularly in the kidneys [331, 332]. Notably, SCFAs modulate miRNA expression that targets these receptors, suggesting a miRNA‐mediated mechanism linking gut microbial metabolism to renal function [325, 333, 334]. In hypertensive individuals, decreased intestinal SCFA absorption and increased fecal SCFA excretion have been observed, accompanied by altered renal miRNA expression patterns [325]. Moreover, SCFAs function as histone deacetylase (HDAC) inhibitors, exerting epigenetic control over genes involved in vascular homeostasis and inflammation [335]. According to previous research, hydrogen sulfide (H_2_S) supplementation restores normotensive phenotypes by modulating the SCFA‐miRNA‐receptor axis in the kidney, offering a potential therapeutic approach targeting both microbial metabolism and host gene regulation [325]. H_2_S was also shown to remodel gut microbiota composition, thereby influencing host miRNA expression and attenuating hypertensive kidney injury [335, 336, 337].

Beyond vascular regulation, ncRNAs also modulate neuroinflammatory pathways relevant to hypertension‐associated organ damage [337, 338, 339]. For instance, miR‐194‐5p inhibits NLRP3 inflammasome activation by disrupting the TRAF6‐NLRP3 interaction, thereby suppressing neuroinflammation [340, 341]. Similarly, miR‐124‐3p alleviates microglial activation and secondary inflammation following basal ganglia hemorrhage through the same axis [342]. Interestingly, gut microbiota‐derived signals may also participate in brain‐kidney‐gut crosstalk during hypertensive crises [343, 344]. In models of intracerebral hemorrhage (ICH), miR‐150‐3p, delivered via mesenchymal stem cell (MSC)‐derived exosomes, has been shown to alter gut microbial composition, including changes in Proteobacteria, Muribaculaceae, Acinetobacte, and Lachnospiraceae_NK4A136_group, and is downregulated in ICH [338, 345, 346]. These findings suggest that microbiota‐regulated exosomal miRNAs actively contribute to the neuroinflammatory milieu and microbial dysbiosis characteristic of hypertension‐related cerebrovascular complications [345]. Collectively, these studies highlight a multifaceted role for gut microbiota‐ncRNA interactions in the pathogenesis of primary hypertension, encompassing vascular, renal, and neuroimmune axes. Targeting the microbiota‐miRNA‐receptor network may represent a promising strategy for the prevention and treatment of hypertension and its complications.

#### 3.2.5 Pulmonary Hypertension

Emerging studies have begun to uncover the involvement of gut microbiota‐regulated ncRNAs in the pathogenesis of pulmonary hypertension, particularly through their influence on pulmonary vascular remodeling under hypoxic conditions [347, 348, 349]. One such regulatory axis involves miR‐208a‐3p, a microRNA significantly downregulated in lung tissues exposed to hypoxia. Interestingly, preconditioning with the symbiotic gut bacterium *Bacillus mucilaginosus* (*B. mucilaginosus*) restores miR‐208a‐3p expression, suggesting a microbiota‐mediated protective mechanism in pulmonary arterial hypertension (PH) [350]. Functionally, miR‐208a‐3p mimics have been shown to enhance hypoxia‐induced aberrant proliferation of human pulmonary arterial smooth muscle cells (hPASMCs) by promoting cell cycle progression. In contrast, knockdown of miR‐208a‐3p in hypoxia‐induced PH mouse models abolishes the protective effects of *B. mucilaginosus* preconditioning, further supporting the causal role of this miRNA in vascular remodeling [350, 351, 352]. Mechanistically, miR‐208a‐3p directly targets the 3’‐UTR of neuro‐oncological ventral antigen 1 (NOVA1) mRNA, a gene implicated in post‐transcriptional regulation of alternative splicing. Hypoxia significantly induces NOVA1 expression in lung tissues, which is reversed following *B. mucilaginosus* treatment. Silencing NOVA1 suppresses hypoxia‐induced hPASMC proliferation by modulating the cell cycle, thereby recapitulating the protective effects of microbiota‐mediated miR‐208a‐3p regulation. Taken together, these findings delineate a novel gut‐lung axis in the pathophysiology of pulmonary hypertension, in which *B. mucilaginosus* exerts its beneficial effects by restoring miR‐208a‐3p expression and inhibiting NOVA1‐driven vascular remodeling [350, 353, 354, 355]. The miR‐208a‐3p/NOVA1 axis thus represents a promising therapeutic target in hypoxia‐associated pulmonary vascular diseases. These data are derived primarily from preclinical models, and whether similar *B. mucilaginosus*‐miR‐208a‐3p‐NOVA1 circuits operate in human pulmonary hypertension remains to be determined.

### 3.3 Neurological Disorders

#### 3.3.1 Depression

Emerging evidence suggests that the gut microbiota communicates bidirectionally with the central nervous system (CNS) via the microbiota‐gut‐brain axis, thereby influencing emotional and cognitive functions [126, 356, 357]. This interaction involves immunological, neuroendocrine, and neuronal pathways. Microbial‐derived signals can modulate peripheral immune cell activity, promote cytokine release into systemic circulation, and alter gastrointestinal function, all of which may affect brain physiology [358, 359]. Additionally, microbial dysbiosis can activate central immune cells such as microglia, whose overactivation leads to excessive production of pro‐inflammatory cytokines, initiating neuroinflammatory cascades—a key pathological feature in major depressive disorder (MDD) [360, 361, 362]. The gut microbiota also exerts neuroactive effects by modulating enteroendocrine and enterochromaffin cells through microbial metabolites such as secondary bile acids, indole derivatives, and SCFAs, which regulate the secretion of neuropeptides and neurotransmitters, including glucagon‐like peptide‐1 (GLP‐1) and 5‐hydroxytryptamine (5‐HT) [363, 364, 365]. Moreover, specific gut microbes are capable of synthesizing and secreting monoamine neurotransmitters that are closely associated with mood regulation [366, 367, 368, 369]. For instance, *Candida*, *Streptococcus*, *Escherichia*, and *Enterococcus* can produce 5‐HT [370, 371]; *Bacillus* and *Serratia* are known to synthesize dopamine; *Escherichia*, *Bacillus*, and certain *Yeast* strains generate norepinephrine [370]; *Lactobacillus* species are capable of producing acetylcholine; while both *Lactobacillus* and *Bifidobacterium* can secrete γ‐aminobutyric acid (GABA) [372]. Among these, 5‐HT plays a central role in the pathophysiology of depression, and its decreased levels are strongly associated with depressive symptoms [373, 374]. The gut microbiota regulates 5‐HT biosynthesis in part by modulating the tryptophan metabolic pathway [375, 376]. These capacities have been demonstrated primarily in vitro and in animal models, and while they illustrate how specific taxa could influence neuroactive signaling, the extent to which individual species causally shape depressive phenotypes in humans remains unclear.

In parallel, pathological alterations in the enteric nervous system (ENS) exacerbate depressive phenotypes by impairing intestinal motility, permeability, immune defense, and secretory function [377, 378]. Psychological stress may be transmitted through the ENS to intensify intestinal inflammation, while persistently elevated glucocorticoid levels have been shown to impair the transcriptional maturity of enteric neurons, promote monocyte recruitment, and contribute to dysmotility [379]. Furthermore, microbial metabolites, regulatory hormones, and immune mediators interact with enteric neurons, vagal afferents, and spinal sensory nerves innervating the gut [380, 381, 382]. These peripheral signals are transmitted to brain regions involved in cognition, emotion, somatosensation, and feeding behavior, thereby modulating CNS function [383, 384]. Conversely, efferent signals from the CNS, particularly via vagal and spinal nerves, can influence gut homeostasis and microbial composition through top‐down regulation of ENS activity [385]. This bidirectional communication not only coordinates gastrointestinal physiology and mucosal immunity but also shapes ncRNA expression profiles, highlighting the pivotal role of gut microbiota‐ncRNA crosstalk in the development and progression of depression [386].

#### 3.3.2 Myalgic Encephalomyelitis/Chronic Fatigue Syndrome

Myalgic encephalomyelitis/chronic fatigue syndrome (ME/CFS) is a complex, multisystemic disorder characterized by persistent fatigue, cognitive dysfunction, and immune dysregulation [387, 388]. Accumulating evidence suggests that dysbiosis of the gut microbiota plays a critical role in the pathogenesis of ME/CFS [389]. Patients with ME/CFS often exhibit significant alterations in gut microbial composition, notably reduced microbial diversity and shifts in the abundance of specific taxa [390, 391, 392, 393]. For instance, a marked reduction in *Enterococcus faecalis Przewalskii*, a key butyrate‐producing bacterium with anti‐inflammatory properties, has been observed in ME/CFS patients, and its depletion correlates inversely with fatigue severity [394]. Moreover, compositional changes include decreased relative abundance of the phyla *Firmicutes* and *Actinobacteria*, accompanied by an increase in *Cyanobacteria* and *Ascomycota*, suggesting a disturbed microbial ecosystem [395, 396]. These microbial alterations are thought to contribute to aberrant immune activation and chronic low‐grade inflammation, as evidenced by elevated circulating levels of pro‐inflammatory cytokines such as IL‐1β, IL‐6, and TNF‐α in ME/CFS patients [395]. Such cytokine profiles are closely associated with gut dysbiosis and may contribute to the characteristic symptomatology of ME/CFS, including fatigue, pain, and neuroinflammation [397, 398]. Although the precise mechanisms remain to be fully elucidated, emerging studies suggest that microbiota‐derived metabolites and microbiota‐regulated ncRNAs may modulate host immune responses and energy metabolism, thereby influencing ME/CFS pathophysiology [399, 400]. In this context, targeted modulation of the intestinal microbiota—through probiotics, prebiotics, FMT, or dietary interventions—has been proposed as a promising therapeutic avenue for alleviating ME/CFS symptoms and restoring immune and metabolic homeostasis.

#### 3.3.3 Alzheimer's Disease

Alzheimer's disease (AD) is a progressive neurodegenerative disorder characterized by cognitive decline, neuronal loss, and neuroinflammation [401, 402]. Emerging evidence suggests that the gut‐brain axis, particularly gastrointestinal inflammation and barrier dysfunction, plays a pivotal role in the pathogenesis of AD [403, 404, 405]. Gastrointestinal damage, often resulting from dysbiosis or indigestion, can trigger local inflammation and compromise intestinal barrier integrity [406, 407]. This disruption exposes the ENS to chronic inflammatory insults, promoting the misfolding and aggregation of α‐synuclein, a hallmark protein involved in neurodegeneration [408, 409, 410]. Misfolded α‐synuclein is transported retrogradely to the brain via the vagus nerve, contributing to early neuropathological changes in AD [411]. Concurrently, sustained gastrointestinal inflammation induces systemic release of inflammatory mediators, including pro‐inflammatory cytokines, immune cells, antibodies, and bacterial endotoxins [412, 413, 414]. These circulating factors can impair the blood‐brain barrier (BBB), facilitating their entry into the CNS. Within the CNS, these mediators activate microglial cells, which initiate and perpetuate a neuroinflammatory cascade [415, 416]. Chronic microglial activation leads to the excessive release of neurotoxic substances and exacerbates neuronal injury and cognitive impairment, accelerating the progression of AD pathology [417, 418]. Although the role of ncRNAs in this gut‐brain inflammatory circuit is still being elucidated, recent studies suggest that dysregulated expression of specific miRNAs and lncRNAs—potentially modulated by gut microbiota—may contribute to microglial activation and amyloid‐β deposition [419, 420, 421]. Thus, targeting gut microbiota and its downstream ncRNA‐mediated signaling may offer novel strategies for preventing or delaying AD onset by restoring intestinal homeostasis and suppressing systemic and neuroinflammation [422, 423, 424].

### 3.4 Immune‐Mediated Diseases

Although Vogt‐Koyanagi‐Harada (VKH) syndrome and experimental autoimmune uveitis (EAU) models have contributed to our understanding of disease mechanisms, limitations remain in fully separating microbiota‐mediated effects from other factors [425, 426]. Several non‐mutually exclusive mechanisms have been proposed for the involvement of the microbiota in the pathogenesis of uveitis and other autoimmune diseases, including (a) antigen mimicry; (b) ecological dysregulation (including HLA‐associated dysbiosis) leading to impaired microbiome‐dependent immune homeostasis; (c) ecological dysregulation resulting in disruption of intestinal barrier function; and (d) migration of intestinal mucosal‐associated lymphocytes to peripheral sites [427, 428]. Certain bacterial antigens within the gut microbiota may structurally resemble host self‐antigens, thereby inducing cross‐reactive immune responses [429, 430]. For example, in the R161H mouse model, intestinal bacterial antigens mimic retina‐specific antigens (e.g., IRBP), activating autoreactive T cells and leading to uveitis [431]. This antigen‐mimicking mechanism may cause the immune system to attack ocular tissues and trigger inflammation mistakenly [432]. Ecological dysregulation may lower the threshold for immune activation by disrupting the Th17/Treg balance and promoting excessive production of pro‐inflammatory cytokines, such as interleukin‐17 (IL‐17) [433, 434, 435]. In antibiotic‐treated mice, an increased frequency of Tregs has been observed in the gastrointestinal lamina propria, as well as in cervical and mesenteric lymph nodes [436]. Tregs play a critical role in immunomodulation by suppressing excessive immune responses and thereby reducing inflammation [437, 438]. Additionally, lymphocytes such as Th17 cells, activated within the gut mucosal immune system in response to microbial stimulation, may migrate to peripheral target organs, such as the eye, via the bloodstream [439, 440]. Upon arrival, these cells secrete pro‐inflammatory cytokines that exacerbate the pathological process of uveitis. These findings suggest that the gut microbiota may influence the severity of EAU by regulating the number and activity of Tregs [88].

### 3.5 Musculoskeletal Disorders

Part of the impact of microbial dysbiosis on bone healing and overall bone health is mediated by the gut microbiota, which facilitates the trafficking of TNF^+^ T cells and Th17 inflammatory cells to the bone marrow and modulates the systemic inflammatory state—an emerging concept referred to as the “brain‐gut‐bone” axis [441, 442]. Inflammatory cells are recruited to the site of injury by growth factors and chemokines, initiating the formation of an extracellular matrix that gives rise to a fibrous bone scab [443]. This scab, considered a form of woven or immature bone, is structurally weaker than mature bone but serves as a temporary scaffold for future periosteal ossification occurring proximal and distal to the fracture site. Following the resolution of acute inflammation, MSCs differentiate into osteoblasts and initiate periosteal ossification. This process forms a continuous thin layer of bone between the underlying healthy bone or cartilage and the fibrous scab, gradually replacing or reinforcing it [444, 445, 446]. Crucially, successful bone healing requires a balanced interaction between osteoclast and osteoblast activity [447]. The gut microbiota contributes to bone formation by modulating insulin‐like growth factor 1 (IGF‐1) production [448, 449, 450]. Dysbiosis has been associated with impaired bone homeostasis through disrupted nutrient absorption, including calcium and vitamin D, and altered regulation of osteoclast function [451, 452]. For instance, *Bacillus pumilus*, a beneficial gut bacterium, is commonly depleted in individuals with inflammatory skin disorders and osteoporosis [453, 454]. Its reduction correlates with heightened systemic inflammation and immune dysregulation, highlighting its protective role in bone and systemic health [455].

### 3.6 Infectious Diseases

Severe respiratory viral infections provide a compelling context in which the gut microbiota can shape immune outcomes at distal mucosal sites. Through systemic immune modulation, intestinal microbes may influence pulmonary responses and engage ncRNA networks that fine‐tune host defense. In a murine model, maternal supplementation with *Lactobacillus* altered the offspring's gut microbiota and conferred protection against respiratory syncytial virus (RSV) infection by limiting pulmonary inflammation, mucus production, and Th2 polarization, highlighting a role for microbiota in antiviral immune programming [456]. Although this model did not directly implicate miRNAs, clinical studies in RSV‐infected infants have shown that airway miRNA‐mRNA co‐expression networks correlate with disease severity, suggesting that mucosal miRNA signatures may reflect immune status and serve as accessible biomarkers for disease monitoring [457]. Together, these findings support the view that ncRNA dynamics may act both as effectors and readouts of microbiota‐driven immune modulation in respiratory viral infections.

A similar axis may operate in systemic parasitic infections such as malaria, although the mechanistic links are less well defined. Numerous studies have shown that the composition of the gut microbiota correlates with disease severity. Specific bacterial lineages have been associated with increased risk of severe malaria in both clinical and experimental settings, suggesting that microbial ecology may influence host susceptibility and pathophysiological responses to *Plasmodium* infection [458]. On the other hand, clinical and review‐based evidence has identified circulating miRNAs in malaria patients as candidate biomarkers of inflammation and organ damage. While it is unclear whether these miRNAs are directly modulated by the gut microbiota, they provide a feasible molecular window into ncRNA involvement during infection progression [459]. Taken together, although the mechanistic details of the microbiota‐ncRNA axis in malaria are still emerging, existing data supports a link between microbial composition and infection severity and point to miRNAs as potential molecular readouts of inflammatory status and disease trajectory.

In *Listeria monocytogenes* infection, intestinal microRNA responses depend on the presence of gut microbiota, and miR‐146a deficiency enhances resistance by reshaping microbial composition [460, 461]. In *Clostridioides difficile* infection (CDI), fecal microbiota transplantation restores microbial diversity and reprograms circulating and colonic miRNA profiles targeting immune and metabolic pathways, with treatment‐responsive miRNAs proposed as biomarkers [462]. *C. difficile* flagellin induces miR‐27a‐5p via Toll‐like receptor 5 (TLR5) and NF‐κB, and its mimic reduces epithelial inflammation while preserving bacterial clearance in vivo [463]. In *Trichuris muris* infection, fecal small RNA sequencing identifies miRNA signatures linked to fibrosis and barrier repair that correlate with intestinal pathology, supporting a diagnostic role for this axis [464]. These examples indicate that in infectious disease settings, microbiota‐regulated ncRNAs shape infection pathogenesis and clinical course, while also providing non‐invasive readouts and potential targets for microbiota‐directed or RNA‐based interventions.

## 4 Representative Correlative and Causal Microbiota‐ncRNA Interactions

Building on the disease‐specific examples summarized in Section 3, it becomes clear that the current literature on the gut microbiota‐ncRNA axis comprises two qualitatively different types of evidence. In view of the very broad range of conditions potentially influenced by this axis, we do not aim to provide an exhaustive catalogue here. Instead, we highlight representative disease settings in which the available data are sufficiently mature to distinguish predominantly correlative microbiota‐ncRNA alterations from experimentally supported causal interactions. In some contexts, changes in microbiota composition and ncRNA expression are observed in parallel and associate with disease incidence, activity, or prognosis, but without direct proof that the microbiota drives ncRNA reprogramming and the ensuing immune or tumor phenotypes. In other contexts, colonization experiments, dietary or metabolite interventions, fecal microbiota transplantation, and ncRNA gain‐ or loss‐of‐function studies jointly support a causal pathway from defined microbial communities or metabolites to specific ncRNAs and downstream immune dysregulation or tumorigenesis. It is also important to note that, with few exceptions, these causal chains are established primarily in preclinical models or small single‐centre cohorts, and mechanistic validation across large, independent human populations is still lacking. To clarify these different levels of evidence, we summarize in Table 2 a set of representative microbiota‐ncRNA interactions that mirror the major disease entities discussed in Section 3, indicating for each context the key microbial features, the principal ncRNAs involved, the core mechanistic link, and whether the available data are mainly correlative or experimentally supported as causal.

**TABLE 2:** Representative microbiota‐ncRNA interactions in disease.

Disease	Bacterial species	ncRNA	Mechanism	Interaction type	Evidence context	References
IBD	IBD‐associated dysbiosis	miR‐223	Dysbiotic gut microbiota coincide with reduced miR‐223, leading to dysregulated TNFα/NF‐κB/NLRP3 signalling and weakened epithelial barrier integrity via TMIGD1, thereby aggravating colitis.	The immunoregulatory role of miR‐223 is mechanistically well established, but the specific bacterial taxa driving its dysregulation in human IBD remain undefined. **(Correlative)**	In vitro experiment; in vivo experiment (mouse colitis models); clinical cohorts	[118, 119]
RA	RA‐associated dysbiosis	lncRNA MEG3	The MEG3/miR‐93‐5p/SMAD7 axis regulates proliferation and inflammatory activation of synovial fibroblasts in the setting of RA‐associated gut dysbiosis.	The ncRNA‐immune mechanism is well defined, but a direct connection between specific gut taxa and MEG3 regulation has not yet been experimentally demonstrated. **(Correlative)**	In vitro experiment; clinical tissue samples	[120]
Tuberculosis	*Bacteroides fragilis*	lncRNA‐CGB	Commensal *Bacteroides fragilis* upregulates lncRNA‐CGB in CD4^+^ T cells, which binds EZH2, reduces H3K27me3 at the *Ifng* locus and enhances IFN‐γ‐dependent antimycobacterial immunity.	Mouse models establish causality for this axis, while supporting human data remain associative. **(Causal**)	In vitro experiment; in vivo experiment (mouse TB models)	[42]
CRC	*Fusobacterium nucleatum*	miR‐21, miR‐4802, miR‐18a	*F. nucleatum* activates TLR4‐MyD88‐NF‐κB, upregulates miR‐21 and downregulates miR‐4802/miR‐18a, suppressing RASA1/PDCD4 and enhancing ULK1/ATG7, thereby promoting proliferation and chemoresistance.	Experiments support a direct microbiota‐miRNA‐tumor progression pathway, patient cohorts show tumor enrichment with variable effect sizes across populations. **(Causal**)	In vitro experiment; in vivo experiment (mouse/xenograft models); clinical tumor samples	[192, 194, 195]
CRC	*E. coli*	miR‐20a‐5p	*Colibactin* from *E. coli* induces DNA damage and c‐Myc expression, which upregulates miR‐20a‐5p, targets SENP1 and alters p53 SUMOylation to drive a senescence‐associated secretory phenotype and tumorigenesis.	Experimental dissection identifies the c‐Myc/miRNA/SENP1 axis as mediating genotoxin‐induced tumorigenesis, but its prevalence and impact across human populations are still being defined. **(Causal**)	In vitro experiment; in vivo experiment (mouse models); clinical biopsies	[196]
CRC	*Porphyromonas gingivalis*	hsa‐miR‐3943	*Porphyromonas gingivalis* activates inflammasome signalling and alters hsa‐miR‐3943 expression, facilitating colorectal cancer progression and metastasis.	Preclinical models link *P. gingivalis*‐induced miR‐3943 changes to metastatic traits, whereas epidemiological data in CRC patients remain sparse. **(Causal**)	In vitro experiment; in vivo experiment (xenograft models)	[197]
CRC	*Faecalibacterium prausnitzii*	miR‐92a, miR‐203	Butyrate produced by *Faecalibacterium prausnitzii* decreases c‐Myc/miR‐92a and induces miR‐203, leading to p57 upregulation and EMT inhibition, thus suppressing CRC cell proliferation and metastasis.	Butyrate‐miRNA‐tumor suppression is causal in experimental systems; in patients, reduced *F. prausnitzii* is mainly observed as part of broader dysbiosis. **(Causal**)	In vitro experiment; in vivo experiment (xenograft models)	[198, 199, 200]
CRC	Dysbiosis with *Bacteroides fragilis/E. coli*	Circ‐0084615, circSPARC, miR‐599, miR‐485‐3p	Dysbiotic microbiota modulate circ‐0084615 and circSPARC; these circRNAs sponge miR‐599/miR‐485‐3p, regulate DNMT3A and JAK/STAT signaling, and enhance CRC cell proliferation and migration.	Microbiota‐exposed CRC models support the mechanism, the role of defined dysbiotic consortia in patients is still being clarified. **(Causal**)	In vitro experiment; in vivo experiment (mouse models)	[215, 216, 217]
NAFLD	Dysbiotic microbiota from miR‐30a‐5p^−/−^ mice	miR‐30a‐5p	FMT from miR‐30a‐5p knockout donors aggravates hepatic steatosis and arachidonic‐acid pathway activation (COX/LOX, ALOX5/ALOX12), enhancing lipid peroxidation and ferroptosis; miR‐30a‐5p restoration reverses these effects.	Experiments support a causal microbiota‐ncRNA‐steatosis axis, yet specific bacterial drivers and human validation remain undefined. **(Correlative)**	In vivo experiment (mouse FMT models)	[238, 239, 240, 241, 242, 243]
Sepsis	HFD‐altered microbiota; *Staphylococcus spp.; Salmonella enterica*	miR‐222, lncRNA GAS5, miR‐146a	Radiation and HFD reshape gut microbiota and macrophage programs (miR‐222‐GAS5 axis), increase barrier leak and bacterial translocation; pathogen exposure induces NF‐κB‐driven miR‐146a, which targets MyD88/TRIF to form a negative‐feedback loop on TLR signaling.	Preclinical models link microbiota disruption and ncRNA reprogramming to barrier failure and sepsis, whereas key sepsis‐associated taxa and human ncRNA data are still incomplete. **(Correlative)**	In vitro experiment; in vivo experiment (mouse sepsis models)	[253, 254, 255, 256, 257, 258, 259, 260, 261, 262, 263, 264, 265, 266, 267, 268, 269, 270, 271]
Atherosclerosis	Commensal producers of IPA and SCFAs	miR‐142‐5p, miR‐10b, miR‐7a‐5p	Microbial IPA and SCFAs modulate vascular and myocardial miRNAs that control oxidative stress, endothelial function and adverse remodeling, thereby influencing cardiometabolic risk.	There are strong associative and prognostic links in human cohorts, whereas mechanistic microbiota‐ncRNA‐brain circuits are derived mainly from preclinical studies. **(Correlative)**	In vivo experiment (mouse models); clinical cohorts	[303, 304, 305, 306, 307, 308, 309, 317, 318, 319, 320, 321, 322]

## 5 Clinical Applications of Gut Microbiota‐ncRNA Axis

**FIGURE 5:**
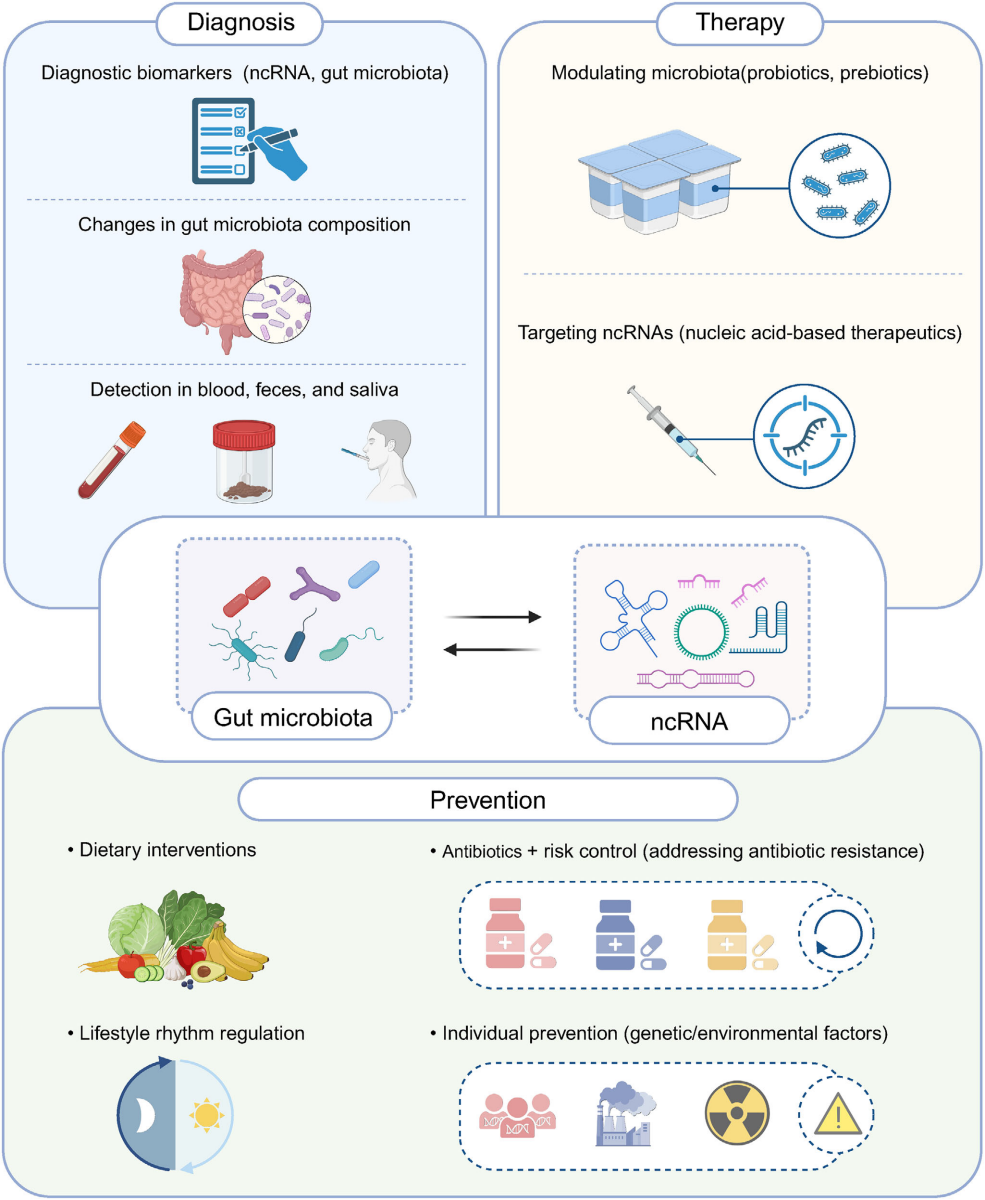
Clinical applications of the gut microbiota‐ncRNA axis. The gut microbiota‐ncRNA axis integrates microbial signals with host immune and epithelial regulatory programs and can be leveraged for diagnostic, therapeutic, and preventive applications. In diagnostics, composite profiles of microbiota‐derived features and ncRNAs serve as minimally invasive biomarkers that reflect microbial dysbiosis and ncRNA‐driven immune perturbation, supporting early detection, disease monitoring, and treatment‐response stratification. In therapeutics, modulation of gut microbiota and targeted correction of dysregulated ncRNAs aim to re‐establish host‐microbe and immune homeostasis in inflammatory and malignant diseases. Preventive strategies, including lifestyle and dietary interventions, circadian rhythm alignment, and judicious antibiotic use, seek to preserve a resilient microbiota‐ncRNA network and reduce long‐term disease risk.

The growing recognition of the gut microbiota‐ncRNA axis as a pivotal regulator of immune homeostasis has opened new avenues for clinical applications, particularly in disease diagnosis, therapeutic interventions, and prevention strategies (Figure 5). The intricate interactions between gut microbes and host ncRNAs offer a unique opportunity to identify novel biomarkers and therapeutic targets, enabling personalized treatment approaches for a variety of diseases. By modulating immune responses through microbial metabolites and ncRNAs, this axis has potential for the development of precision medicine strategies aimed at treating immune‐related disorders, cancers, metabolic diseases, and more. In this section, we explore the clinical application prospects of targeting the gut microbiota‐ncRNA axis, with a focus on diagnostic tools, therapeutic interventions, and preventive measures that hold promise for improving patient outcomes.

### 5.1 Diagnostic Utilities

In recent years, with the deepening understanding of the interaction mechanisms between the gut microbiota and ncRNAs, their clinical potential has garnered increasing attention [277]. In the field of disease diagnosis, the gut microbiota and ncRNAs are gradually being explored as promising biomarkers [212]. In IBD, microbial dysbiosis often disrupts intestinal mucosal homeostasis, promoting inflammatory responses [465, 466]. MiRNAs regulate immune system activation through multiple signaling pathways and have emerged as reliable diagnostic markers for IBD due to their distinct expression profiles in patients compared to healthy controls [467]. Moreover, various lncRNAs have been implicated in IBD [468, 469, 470, 471]. For instance, research has demonstrated that the expression levels of lncRNAs KIF9‐AS1 and LINC01272 were significantly elevated in tissue and plasma samples from IBD patients, whereas lncRNA DIO3OS expression was markedly reduced compared to healthy individuals [472]. Monitoring lncRNA expression changes thus offers the potential for diagnosing and clinically evaluating IBD [28]. Similarly, in gastrointestinal cancers, circRNAs exhibit aberrant expression patterns owing to their stability and tissue‐specific expression, enabling discrimination between malignant and normal tissues [473]. Changes in gut microbiota composition are also highly correlated with disease progression and patient survival, providing valuable prognostic information [474]. Additionally, the gut microbiota's impact on immune responses and metabolism alters fecal metabolites and immune markers, paving the way for the development of non‐invasive diagnostic techniques [475].

Advances in assay technologies have further enhanced the utility of gut microbiota and ncRNAs in disease diagnostics [476, 477, 478]. High‐resolution gene sequencing enables detailed analyses of gut microbial composition and function, identifying disease‐specific microbial signatures [479]. Concurrently, non‐coding RNA expression profiling has become increasingly refined. In cancers, genomic mutations, copy number variations, and epigenetic alterations affect ncDNA and consequently the expression of corresponding ncRNAs, detectable via transcriptional analysis [480, 481]. Bioactivity detection techniques such as quantitative fluorescence PCR, microarrays, and direct sequencing have provided robust platforms for the functional assessment of ncRNAs [482, 483]. Given the stability of ncRNAs in various body fluids, early disease screening and diagnosis can be achieved by measuring ncRNA levels in samples such as blood and saliva, offering deeper insights into their pathological roles [484].

### 5.2 Therapeutic Intervention

In terms of therapeutic interventions, strategies targeting the gut microbiota‐ncRNA axis offer novel approaches for the treatment of immune disorders. The application of probiotics and prebiotics is a common method for modulating the intestinal microbiota [485, 486]. By improving the composition and function of the gut flora, these interventions can influence ncRNA expression and contribute to disease treatment [176]. For instance, in certain intestinal immune disorders such as inflammation, tumors, infections, and allergies, specific probiotics can help rebalance the intestinal microbiota, modulate host immune responses, and attenuate disease symptoms [487]. Additionally, as an emerging therapeutic tool, FMT has demonstrated remarkable efficacy in treating refractory intestinal diseases such as recurrent *Clostridioides difficile* infections [488, 489]. By transplanting gut flora from healthy donors into patients, FMT restores microbial ecological balance, modulates the host immune system, and influences ncRNA expression, improving disease outcomes [462, 490]. While FMT has shown some effectiveness in the management of IBD, its safety and tolerability still require further investigation [491, 492]. The development of nucleic acid‐based therapeutics has further expanded the potential for ncRNA‐targeted interventions. By designing small‐molecule drugs or antisense oligonucleotides, it is possible to regulate the expression of specific ncRNAs and thereby intervene in disease onset and progression [493, 494, 495]. For example, miRNAs can function as mRNA antagonists to inhibit overexpressed genes or as miRNA mimics to restore aberrantly expressed miRNAs [496]. Using a Pickering double emulsion system to encapsulate a miR‐146a‐5p overexpression plasmid, subsequent experiments in an LPS‐induced mouse model of IBD demonstrated that miR‐146a‐5p mitigates intestinal inflammation through inhibition of the TLR4/NF‐κB pathway. miR‐146a‐5p also regulated intestinal homeostasis by targeting RNA polymerase σ‐factor RpoD and α‐galactosidase A, thereby influencing the growth of *Lactobacillus reuteri* [497]. However, to date, these ncRNA‐based therapeutic approaches have only been validated in inducible animal models of IBD, and their clinical efficacy remains to be determined [493]. In the field of cancer therapy, targeted ncRNA strategies against miRNAs and lncRNAs are gaining increasing attention [498, 499, 500]. These strategies affect tumor development and progression by regulating gene expression in tumor cells, modulating immune cell function, controlling pro‐inflammatory cytokine secretion, and maintaining systemic immune homeostasis [501, 502].

### 5.3 Prevention Strategy

In terms of prevention strategies, modulation of the gut microbiota and ncRNAs is considered a crucial approach for preventing the development of immune‐related diseases. Diet is widely recognized as a major factor influencing the gut microbial ecosystem [503]. Gut microbes utilize nutrients from ingested food, release harmful and beneficial metabolites, and modulate the host immune system [504, 505]. Studies have shown that consumption of prebiotic‐rich foods (e.g., dietary fiber, oligosaccharides, inulin) or unsaturated fatty acids promotes the growth and proliferation of beneficial flora, thereby improving gut microbiota composition and function [506, 507, 508]. Additionally, certain dietary components, such as polyphenolic compounds, have been found to influence ncRNA expression, exerting immunomodulatory effects (Table 3) [222, 486, 509, 510, 511, 512, 513, 514, 515, 516, 517, 518, 519, 520, 521]. Lifestyle modifications, including maintaining regular circadian rhythms and adopting time‐restricted feeding patterns, positively affect gut microbiota composition and ncRNA expression [522].

**TABLE 3:** Dietary regulation of the gut microbiota‐ncRNA axis: mechanisms and disease prevention.

Food and component	Targeted microbiota	Related ncRNA	Mechanisms of action	Preventable diseases	References
Ginger	*Lactobacillaceae*	mdo‐miR7267‐3p, ath‐miR167a	a). GELNs rich in PA are selectively taken up by Lactobacilli; b). GELN miRNAs target the ycnE gene in LGG), enhancing tryptophan metabolite IA3A production to activate AHR signaling and IL‐22 secretion, improving gut barrier function; c). ath‐miR167a in GELNs downregulates LGG's SpaC pili gene, reducing mucosal colonization and systemic infection risk.	Colitis	[222]
Garlic	↑*Lachnospiraceae*, ↓*Helicobacter/Escherichia‐Shigella*	han‐miR3630‐5p	a). GENs contain has‐miR3630‐5p that targets TLR4 3’UTR to inhibit TLR4/MyD88/NF‐κB signaling; b). Promotes SCFA biosynthesis to enhance gut barrier function and modulate immune responses.	Colitis	[510]
Leafy Greens		miR156a	a). Plant MIR156a directly targets JAM‐A to reduce monocyte adhesion induced by inflammatory cytokines; b). Stably transported via exosomes in human serum to regulate gene expression.	Cardiovascular Diseases	[511]
Corn	↓*Firmicutes*, ↑*Lactobacillus*, ↓*Ruminococcus*	miR156a, miR167a	a). Corn miRNAs (miR156a/167a) target bacterial genes (PAS‐domain histidine kinase, ABC transporters) to inhibit Firmicutes growth; b). Corn matrix (non‐miRNA components) may selectively promote specific microbiota via carbon source provision.	Obesity, Metabolic Syndrome, CVD	[512]
Tartary Buckwheat	↑*E. coli* ↑*L. rhamnosus*	miR6300, miR482b	a). TBDNs contain miRNAs targeting bacterial genes to regulate physiological processes; b). Enhances SCFA production to maintain gut homeostasis and nutrient absorption.	CVD, Diabetes, Obesity, IBD	[513]
Barley	↑*Prevotella*, ↑*Lactobacillus*, ↑*Bifidobacterium*	miR‐126a, miR‐29a, miR‐26a	a). Downregulates miR‐126a/miR‐29a (targeting IRS‐1/PI3K) and upregulates miR‐26a (targeting GSK3β/PEPCK); b). Increases SCFAs while reducing succinate to improve insulin signaling (IRS‐1/PI3K/Akt).	Obesity, T2DM	[514]
Synbiotics	*L. casei*, *L. acidophilus*, *L. bulgaricus*, *L. rhamnosus*, *Bifidobacterium spp*., *S. thermophilus*	miR‐126, miR‐146a	a). Modulates microbiota composition to increase SCFA producers; b). Reduces serum TNF‐α levels; c). Regulates miR‐126 (improves vascular function) and miR‐146a (anti‐inflammatory).	T2DM	[486]
Quercetin	↑*Glutamicibacter*, ↑*Facklamia*, ↑*Aerococcus*	miR‐26a, miR‐125b, miR‐132	a). Increases microbial diversity (ACE/Chao1 indices); b). Upregulates BDNF; c). Modulates inflammatory miRNAs (↓miR‐26a/125b, ↑miR‐132); d). Reduces Aβ plaques and tau phosphorylation via ERK1/2‐CDK5 inhibition; e). Lowers neuroinflammation markers (GFAP, Iba1).	Alzheimer's Disease (AD)	[515]
Osa‐miR168a‐rich foods (Rice, Watermelon)	↑*Muribaculaceae*, ↑*Akkermansia*, ↑*Bifidobacterium*	osa‐miR168a	a). Upregulates tight junction proteins (claudin‐1, occludin, ZO‐1) and MUC2; b). Activates Nrf2 to enhance antioxidant enzymes (NQO1, HO‐1); c). Inhibits NF‐κB to reduce pro‐inflammatory cytokines; d). Directly promotes probiotic growth (e.g., *Bifidobacterium*) via osa‐miR168a.	DSS‐induced Colitis	[516]
Betaine (Spinach, Shellfish, Whole Grains)	↑*A. muciniphila*, ↑*Lactobacillus*, ↑*Bifidobacterium*	miR‐378a	a). Improves HFD‐induced dysbiosis, increasing SCFA producers (*A. muciniphil*a, *Ruminococcus*, *Oscillospira*); b). Acetate/butyrate modulate host miR‐378a promoter methylation; c). miR‐378a targets YY1 to reduce lipogenesis and promote BAT activation/WAT browning; d). Enhances GPR43/41 expression to suppress pro‐inflammatory cytokines.	Obesity, NAFLD, T2DM	[517]
Cherry Juice	↑Beneficial taxa (*Defluviitaleaceae, Myxococcales*, *Dubosiella*), ↓*Enterobacteriaceae*	miR‐200c‐3p, miR‐223‐3p, miR‐132‐3p, miR‐125a‐5p	a). Increases SCFA production; b). Reduces IL‐6, TNF‐α, and LPS levels to improve gut barrier; c). Modulates miRNAs targeting ZEB1/ZEB2 to suppress adipogenesis and inflammation.	HFD‐induced Obesity, Metabolic Disorders	[518]
Pu‐erh Tea (Theabrownin)	↑*Bacteroides*, ↑*Lactobacillus*, ↑*Bifidobacterium*, ↓*Pseudomonas*	miR‐125b‐5p, miR‐223‐3p.R+2, miR‐148b‐3p, miR‐247‐5p	a). Improves Firmicutes/Bacteroidetes ratio; b). Enhances SCFA production; c). Downregulates pro‐inflammatory miRNAs (miR‐125b‐5p, miR‐223‐3p.R2); d). Modulates glucose/lipid metabolism genes (SREBP‐1C, PEPCK, PGC‐1α); e). Antioxidant effects via SOD/CAT/GSH‐Px activation.	Metabolic Syndrome	[519]
Sargassum fusiforme Polysaccharides (SFPs)	↑*Firmicutes/Bacteroidetes ratio*, ↑*Lachnospiraceae* NK4A136_group, ↓*Proteobacteria*	miRNA‐92a‐3p	a). Microbiota‐driven miRNA‐92a‐3p upregulation inhibits Notch1‐Hes1 signaling via 3’UTR binding; b). Promotes goblet cell differentiation and Muc2 secretion; c). Upregulates tight junction proteins (ZO‐1, occludin, claudin‐1); d). Modulates cytokines (↓IL‐1β/IL‐6/TNF‐α/IL‐17, ↑IL‐10/IL‐22).	Citrobacter rodentium‐induced Colitis	[520]
High‐Amylose Maize Starch (HAMS)	↑*R. bromii*, ↑*Turicibacteraceae*, ↓*Peptostreptococcaceae*	miR17, miR19a, miR20a, miR92a	a). Increases butyrate to suppress oncogenic miR17‐92 cluster; b). Reduces protein fermentation products (BCFAs, indole, p‐cresol); c). Shifts microbial metabolism from proteolytic to saccharolytic.	CRC	[521]
Butyrylated HAMS (HAMSB)	↑*Parabacteroides*, ↓*Proteobacteria* (*Bilophila*, *Sutterella*), ↓*Peptostreptococcaceae*	miR17‐92, miR21	a). Direct butyrate release inhibits miR17‐92 cluster and miR21; b). 16‐fold higher fecal butyrate vs. HAMS; c). Reduces pro‐inflammatory bacteria.	CRC	[521]
High‐Amylose Potato Starch (HAPS)	↑*Bifidobacteriales*, ↑*Lactobacillaceae*, ↓*Sutterella*	miR17‐92	a). Suppresses miR17‐92 cluster; b). Reduces proteolytic metabolites (e.g., indole); c). Increases total SCFAs.	CRC	[521]

Pharmacological prophylaxis also plays a vital role in the prevention of immune diseases [523, 524]. The judicious use of antibiotics and immunomodulators can modulate gut microbiota composition and ncRNA expression, thereby reducing disease risk [525, 526]. In a 2,4‐dinitrobenzene sulfonic acid (DNBS)‐induced colitis mouse model, a study found that tetracyclines with antibiotic and immunomodulatory properties (e.g., doxycycline, minocycline, tigecycline) improved gut microbiota composition and function, decreased miR‐223 expression, and increased miR‐142 and miR‐375 expression. These molecular changes enhanced mucosal protection, attenuated inflammatory responses, and improved intestinal barrier function, thereby contributing to the prevention of acute intestinal inflammatory diseases and showing potential therapeutic value for IBD [526].

### 5.4 Barriers to Clinical Translation

Despite the growing promise of microbiota ncRNA‐based approaches, several barriers continue to limit their translation into clinical practice. For diagnostic applications, substantial inter‐individual variability in both microbial composition and ncRNA profiles hampers the development of standardized and reproducible diagnostic criteria [527]. While fecal samples are ideal for combined genomic, epigenomic, and microbiota marker screening, current testing methodologies require further improvement in sensitivity and specificity to ensure diagnostic accuracy [484]. Additionally, for complex diseases such as cancer and cardiovascular disease, single‐tissue biomarkers may not adequately capture the full disease picture, highlighting the need to develop multi‐marker panel approaches for more comprehensive diagnostic evaluation [528].

Significant challenges also remain in therapeutic development. Delivery systems for nucleic acid‐based therapies continue to face limitations related to stability, specificity, and tissue tolerance, all of which restrict their clinical utility. [529]. Despite the great potential of gut microbiota‐ncRNA axis‐based therapeutic strategies for clinical applications, several challenges persist. First, the safety and efficacy of these interventions must be validated through large‐scale clinical trials [530]. Secondly, inter‐individual differences in gut microbiota composition lead to varied epigenetic effects and treatment responses, underscoring the need for personalized therapeutic approaches [527]. Moreover, for complex chronic diseases such as parasite‐induced hepatic fibrosis, single‐treatment strategies may be insufficient, necessitating the development of combinatorial therapeutic regimens [531].

Preventive applications encounter similar obstacles. Potential drug‐related adverse effects and the risk of promoting antibiotic resistance must be carefully evaluated when designing microbiota or ncRNA‐targeted preventive interventions. When developing prevention strategies, personalized prevention programs should also take into account individual genetic background, lifestyle factors, and environmental exposures [532]. Furthermore, future research may explore how to integrate discoveries from the gut microbiota‐ncRNA axis with emerging technological tools such as artificial intelligence to develop more precise and individualized preventive strategies.

## 6 Current Challenges and Future Directions

### 6.1 Recent Achievements

Over the past decade, significant progress has been made in elucidating the gut microbiota‐ncRNA‐immune axis. Methodologically, the advent of high‐throughput sequencing, multi‐omics integration, and single‐cell as well as spatial transcriptomics has provided unprecedented resolution for dissecting microbial, transcriptional, and immunological interactions [533, 534, 535]. Mechanistically, several landmark studies have identified specific microbial taxa, such as *F. nucleatum* and *F. prausnitzii*, that modulate host immunity through the regulation of ncRNAs, including miR‐21, miR‐223, and others [192, 193]. These findings have not only confirmed the functional relevance of microbiota‐derived signals in immune homeostasis but also revealed ncRNAs as critical mediators that link microbial metabolites to immune cell differentiation and effector functions. Clinically, exploratory studies have demonstrated the potential of microbiota signatures and ncRNA profiles as diagnostic and prognostic biomarkers in colorectal cancer, cardiovascular disorders, and neuroinflammatory diseases [536, 537]. Together, these achievements have laid a solid foundation for positioning the gut microbiota–ncRNA axis as both a mechanistic framework for understanding immune regulation and a promising avenue for clinical translation.

### 6.2 Outstanding Limitations

Despite remarkable progress in delineating the gut microbiota‐ncRNA‐immune axis, the field confronts substantial limitations that hinder a transition from associative observations to mechanistic understanding and clinical application. A fundamental challenge is that most existing studies have primarily established correlations rather than causality in human cohorts, making it difficult to delineate direct regulatory pathways [469]. While the bidirectional relationship between the microbiota, ncRNAs, and immunity is well‐established in theory, direct mechanistic evidence from experimental models is still limited [538, 539]. The complete chain linking specific microbial taxa, their metabolites, ncRNA regulators, and immune cell functions is still largely fragmented. Moreover, functional validation of ncRNA targets in distinct immune cell subsets has not been systematically addressed, leaving substantial gaps in our understanding of how microbial signals are translated into precise immunological outcomes.

Translating these discoveries into clinical practice faces equally daunting hurdles. The development of reliable biomarkers based on microbial or ncRNA signatures is impeded by a lack of standardized assays and validation across diverse populations. Therapeutic strategies, particularly those involving ncRNAs, are constrained by challenges in stability, targeted delivery, and potential off‐target effects [540, 541]. Similarly, microbiota‐targeted interventions are hampered by inconsistent long‐term efficacy and the absence of precision designs tailored to individual microbial and genetic backgrounds. Collectively, these limitations underscore the imperative for deeper mechanistic insights and more rigorous translational frameworks.

### 6.3 Future Opportunities

To advance the field, the immediate priority must be to elucidate the precise mechanisms of the gut microbiota‐ncRNA‐immune axis. This will require moving beyond observational studies by strategically deploying precision tools such as CRISPR‐based screening in gnotobiotic models and the use of synthetic microbial communities to definitively link specific microbial signals to ncRNA expression. Simultaneously, single‐cell and spatial multi‐omics technologies will clarify how microbial metabolites regulate ncRNA networks within specific tissue and immune cell environments.

Building on this mechanistic foundation, the field must strive to construct holistic, cross‐system models that explain how gut‐derived signals coordinate physiological and pathological responses across distant organs. Achieving this systems‐level understanding hinges on the sophisticated integration of multi‐omics data, powered by artificial intelligence, to decode the complex communication networks linking the gut to the circulatory, nervous, and immune systems. This integrated knowledge is the essential bedrock for overcoming the critical translational challenges of biomarker standardization and therapeutic development. The path forward lies in creating multimodal biomarker panels that combine microbial, metabolic, and ncRNA signatures, and in pioneering novel delivery systems, such as engineered nanoparticles or bacterial vectors, to overcome the stability and targeting hurdles of ncRNA‐based therapies [542, 543]. Ultimately, the culmination of these efforts should be the development of truly personalized intervention strategies that consider an individual's unique microbial baseline and genetic makeup, ensuring that the promise of the gut microbiota‐ncRNA axis is realized as effective, safe, and precise clinical applications.

## 7 Conclusion

The role of the gut microbiota‐ncRNA axis in immune homeostasis has emerged as a prominent research focus in immunology and microbiology in recent years. This review systematically summarizes the underlying mechanisms by which this axis regulates immune function and highlights its potential clinical applications, underscoring its pivotal role in maintaining immune homeostasis and influencing disease development. At the mechanistic level, the gut microbiota, through its metabolites and immunomodulatory activities, can significantly modulate the host's non‐coding RNA expression profiles, thereby regulating the differentiation, activation, and function of immune cells. The gut microbiota‐ncRNA axis plays a crucial role across multiple biological systems. For example, in immune‐mediated diseases such as VKH syndrome and EAU, this axis contributes to disease progression through antigen mimicry and ecological dysregulation. In the locomotor system, the gut microbiota influences bone healing and the maintenance of bone health by modulating the balance between osteoclast and osteoblast activity. This complex interaction network not only preserves immune homeostasis under physiological conditions but also critically impacts the development of various diseases. However, many aspects of the regulatory mechanisms underlying the gut microbiota‐ncRNA axis remain unclear, including how specific microbial taxa influence the expression of particular ncRNAs and the precise immune cell targets of these ncRNAs, necessitating further investigation.

Clinically, the combined detection of gut microbiota and ncRNA profiles offers promising avenues for early disease diagnosis. Nevertheless, establishing standardized diagnostic biomarkers remains challenging due to substantial inter‐individual variability in microbiota composition and ncRNA expression. Moreover, therapeutic interventions based on this axis—such as probiotics, fecal microbiota transplantation, and nucleic acid‐based therapies—have shown encouraging results in animal models, but clinical validation regarding safety, efficacy, and the stability of nucleic acid drug delivery systems is still required. Future research should emphasize integrated multi‐omics approaches to comprehensively elucidate the mechanisms governing the gut microbiota‐ncRNA axis and explore personalized therapeutic strategies. In conclusion, the gut microbiota‐ncRNA axis plays a vital regulatory role in immune homeostasis and has significant potential for clinical application. However, further in‐depth studies are needed to overcome the current challenges. Priorities for future research should be detailed mechanistic investigations, the development of standardized diagnostic markers, and the exploration of personalized therapeutic strategies. These efforts will advance the field and provide robust support for clinical practice.

## Author Contributions

B.C., G.C., and X.L. researched the literature and drafted the manuscript. Q.L., W.W., W.W., and Z.L. contributed to data collection and figure preparation. Z.Z., X.Z., W.K., and F.R. provided critical revisions and domain‐specific insights. Y.Z. and J.C. conceived and supervised the project. All authors discussed the content and contributed to the final version of the manuscript.

## Conflicts of Interest

The authors declare no conflicts of interest.

## Data Availability

The authors have nothing to report.
